# The relation between implicit statistical learning and proactivity as revealed by EEG

**DOI:** 10.1038/s41598-023-42116-y

**Published:** 2023-09-22

**Authors:** Dorota Sznabel, Rüdiger Land, Bruno Kopp, Andrej Kral

**Affiliations:** 1 Department of Experimental Otology, Hannover Medical School, Hannover, Germany; 2 Cluster of Excellence “Hearing4all”, Hannover, Germany; 3 Department of Neurology, Hannover Medical School, Hannover, Germany

**Keywords:** Neuroscience, Cognitive neuroscience, Learning and memory

## Abstract

Environmental events often occur on a probabilistic basis but can sometimes be predicted based on specific cues and thus approached proactively. Incidental statistical learning enables the acquisition of knowledge about probabilistic cue-target contingencies. However, the neural mechanisms of statistical learning about contingencies (SL_C_), the required conditions for successful learning, and the role of implicit processes in the resultant proactive behavior are still debated. We examined changes in behavior and cortical activity during an SL_C_ task in which subjects responded to visual targets. Unbeknown to them, there were three types of target cues associated with high-, low-, and zero target probabilities. About half of the subjects spontaneously gained explicit knowledge about the contingencies (contingency-aware group), and only they showed evidence of proactivity: shortened response times to predictable targets and enhanced event-related brain responses (cue-evoked P300 and contingent negative variation, CNV) to high probability cues. The behavioral and brain responses were strictly associated on a single-trial basis. Source reconstruction of the brain responses revealed activation of fronto-parietal brain regions associated with cognitive control, particularly the anterior cingulate cortex and precuneus. We also found neural correlates of SL_C_ in the contingency-unaware group, but these were restricted to post-target latencies and visual association areas. Our results document a qualitative difference between explicit and implicit learning processes and suggest that in certain conditions, proactivity may require explicit knowledge about contingencies.

## Introduction

Statistical learning refers to the incidental acquisition of environmental statistics^1–4^, including temporal regularities^5,6^ and spatial distributions^7,8^. Not all regularities are equally relevant for behavior. For example, learning about stimulus occurrence frequency enables different behavioral adaptations than learning about transitional probabilities between stimulus pairs. In the case of learning about predictive relations between temporally separated cue and target events (i.e., statistical learning about contingencies, SL_C_), the greatest behavioral benefit is becoming able to act proactively, e.g., to prepare for the likely upcoming targets upon seeing a target cue^9–11^. Such proactive exploitation of contingencies involves anticipatory motor preparation, substantially shortening response times (RTs) to targets^12–16^.

Exposure to structured input may lead to explicit (declarable) knowledge about the underlying regularities^17^, and typically such knowledge can be flexibly applied in a goal-oriented manner^18^. However, even without explicit knowledge, neural and behavioral responses to statistically structured inputs may—upon sufficient exposure—deviate from those elicited by unstructured inputs in a process referred to as implicit learning^19^. Despite ample evidence that implicit acquisition of statistical regularities affects behavior^4,7,19–22^, the range of adaptations it enables is controversial^18,20,23,24^. In particular, the emergence of anticipatory motor preparation in SL_C_ tasks has rarely been directly addressed^25–27^. Most evidence for implicit statistical learning in humans comes from paradigms that preclude proactive behavior (at least 80% according to a recent review^2^) and instead assess learning with post-exposure memory tests^28^ such as familiarity judgments^5,22,29,30^, or completion and exclusion tasks^21,31–33^. Furthermore, even measures based on RTs are usually inconclusive in showing cue-triggered anticipation^26^.

Anticipatory and preparatory processes can be conclusively demonstrated by measuring EEG brain responses to cue events, such as the contingent negative variation (CNV)^13,15,33^. The CNV is a negative potential occurring over central scalp locations during the interval separating a target cue from an action-requiring target stimulus, commonly interpreted as a marker of anticipation and action preparation^15,34,35^. There are some reports of implicit CNV generation^36–38^; however, these studies construe ‘implicitness’ as the lack of explicit information about the regularities (i.e., ‘incidentalness’), which should not be equated with the absence of contingency awareness. Contingency awareness may emerge spontaneously during learning^17,39,40^. Furthermore, these studies explore the learning of *temporal* aspects of sequences rather than the learning of probabilistic relations between different event types, which may be subserved by distinct neurocognitive mechanisms^2^. Incidental learning of cue-target contingencies has also been shown to influence the late parietal cue-P300 potential^40,41^. Interestingly, the relation of cue-P300 to behavioral indices of learning is less clear than CNV’s^40–42^. While CNV is strictly related to the RT speed-up^12,43,44^, the cue-P300 has been proposed to index the acquisition of knowledge about regularities independently of its behavioral expression^4,41^.

Finally, the brain networks involved in proactivity and their relation to contingency awareness are yet to be established. Prior fMRI studies provided convergent evidence for hippocampal activation during the cue-target interval specific to contingency-aware subjects^39,45,46^. In this context, the hippocampus showed functional connectivity with the posterior medial frontal, anterior cingulate, and midcingulate cortex^39^. Widespread activation of frontal, occipital, and parietal regions during incidental learning of cue-target contingencies was also shown by EEG source reconstruction^40^, although without disambiguating the neural sources of cue-P300 and CNV—two distinct stages of cue processing.

In this study, we asked whether anticipatory motor preparation subserving proactivity can be achieved implicitly, i.e., without contingency awareness. To that end, we examined multiple aspects of behavior and the brain regions involved in cue and target processing. We also asked how these neural correlates correspond to behavioral indices of learning. All analyses were done separately in contingency-aware and unaware subjects to reveal potential differences in implicit and explicit processing.

**Figure 1:**
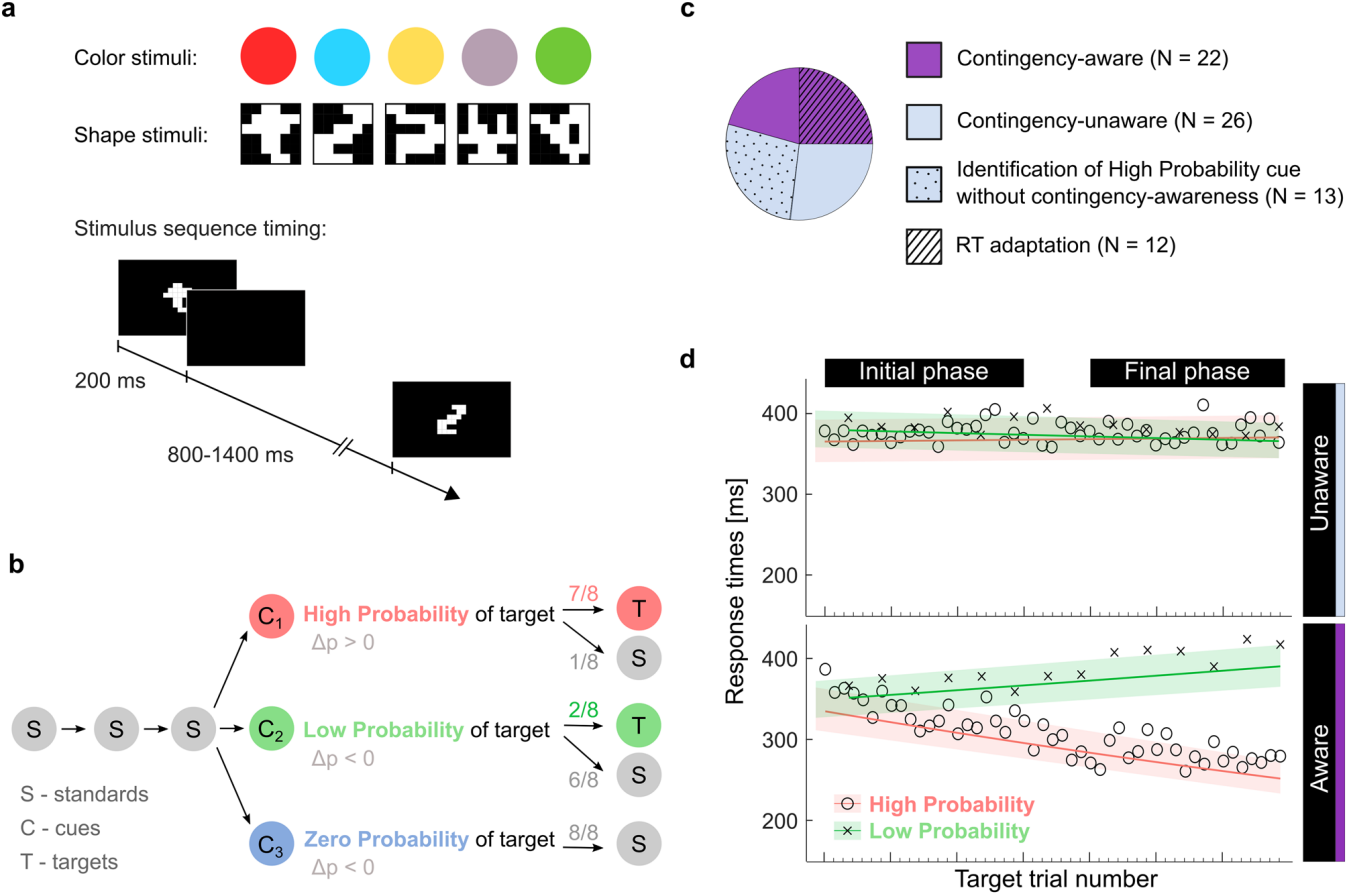
Statistical learning paradigm and behavioral outcomes. (**a**) Stimuli and stimulus sequence timing in the oddball tasks. (**b**) Schematic of stimulus transitions in the oddball-SL task; three cues associated with different target probabilities defined three within-task conditions. (**c**) Classification of individual subjects based on the questionnaire and RTs. (**d**) RTs in the oddball-SL task: data points represent group averages of measured RTs for subsequent targets. LMM-derived estimates are shown in color: RT slopes over trials and 95% confidence intervals.

Forty-eight young adults performed two oddball tasks with five visual stimuli (Fig. 1a), including rare targets, frequent standards, and three rare deviants. One task allowed for statistical learning of cue-target contingencies (oddball-SL task)^41^ because the three deviants served as target cues and were associated with high-, low-, and zero probability of transition into a target (Fig. 1b). Immediately after the oddball-SL task, we ran a questionnaire-based assessment of explicit knowledge about cue-target contingencies and divided subjects into contingency-aware and unaware. In the other task (oddball-control), the stimulus sequence was random, rendering the targets unpredictable. Both tasks were performed under speed pressure. We first focused on the oddball-SL task outcomes, where the main analyses included RT data, cue-elicited event-related potentials (ERPs), and the relation between those ERPs and RT. We expected learning to induce a significant drop in RTs to High relative to Low Probability targets. The cue-P300 and CNV were regarded as neural indices of learning. To substantiate the functional role of cue-ERPs in anticipatory motor preparation, we probed their single-trial relation to the RTs. Behavioral optimization combined with neural markers of anticipation in contingency-unaware subjects would demonstrate that proactivity can be achieved through implicit SL_C_. The brain sources of the ERPs were estimated based on independent component analysis and equivalent dipole source localization. Next, we compared the P300 response to predictable and random targets (the latter derived from the oddball-control task). Depending on whether the previous measures revealed proactivity, this step would elucidate either a proactive or reactive aspect of target processing and its relation to contingency awareness.

## Results

### Learning-related adaptation of RTs is associated with contingency awareness

The participants had to perform a response-time task with visual stimuli, identifying a target among several stimuli with hidden statistical cues. Directly after the task, we used a questionnaire to probe the emergence of explicit knowledge (contingency awareness) about cue-target contingencies. We then assessed the impact of this awareness on behavioral performance and response times.

Although participants were not informed about the cue-target contingencies, 46% of them (N = 22) noticed the predictive relation between the High Probability cue and the target (‘aware’ group; 12 females, mean age = 23.5, SD = 4.2; Fig. 1c). Twenty-six subjects remained unaware of the contingencies (‘unaware’ group; 14 females, mean age = 25.6, SD = 4.9), but after claiming that the stimuli occurred in a random order (see the questionnaire structure, Supp. Fig. S11), 50% of them correctly guessed which stimulus appeared most often before the target (chance level = 25%).

Both groups showed ceiling performance, i.e., hit rates were close to 100% (Supp. Table S19) and did not differ significantly between groups (Wilcoxon rank-sum test; U(22, 26) = 553.5, z = 9 0.317, *p* = 0.751). The false alarm rate was higher for the contingency-aware than the contingency-unaware group (Wilcoxon rank-sum test: U(22, 26) = 519, z = 12–2.4372, *p* = 0.015), although in both groups the false alarm rate was lower than 1% (Supp. Table S19).

Only the contingency-aware group showed behavioral evidence of learning, i.e., a decrease in RTs specific to the predictable targets (Fig. 1d). In the contingency-unaware group, the RTs between targets preceded by High and Low Probability cues did not differ (see Table 1 for ANOVA results on the linear mixed-model (LMM) data, and Supp. Tables S1–S3 for a comprehensive report on the LMM). Hence, the contingency-unaware group did not optimize behavior during the task, but the exposure might have implicitly affected their questionnaire answers.

**Table 1:** 3-way ANOVA (type III sums of squares) on the LMM estimates of response times.

	Sum Sq	Num. df	Den. df	*F*	*p*
Condition	0.07	1	102.6	2.90	0.092
Trial	0.41	1	2739.1	17.01	< 0.001
Group	0.09	1	56.7	3.80	0.056
Condition:Trial	1.21	1	2739.9	50.95	< 0.001
Condition:Group	0.00	1	102.6	0.04	0.840
Trial:Group	0.23	1	2739.1	9.76	0.002
Condition:Trial:Group	2.06	1	2739.9	86.38	< 0.001

Condition (High-/Low-/Zero Probability); Group (Aware/Unaware); Trial—continuous variable based on event latencies.

Complementary to the questionnaire-based classification, we performed subject classification based on individual subjects' RTs. This analysis confirmed the adaptation of RTs in 12 subjects, all of whom belonged to the contingency-aware group (Supp. Table S18 and Fig. S8). Hence, the online behavioral adaptation was present only in the contingency-aware subjects, both on group and individual levels.

### SL_C_ modulated neural responses to cues in contingency-aware subjects

Next, we analyzed the effect of statistical learning on neural responses throughout the experiment. Specifically, we compared the presence and change in P300 and CNV potentials to the different cues in subjects who became aware of the contingencies and those who remained unaware.

**Figure 2:**
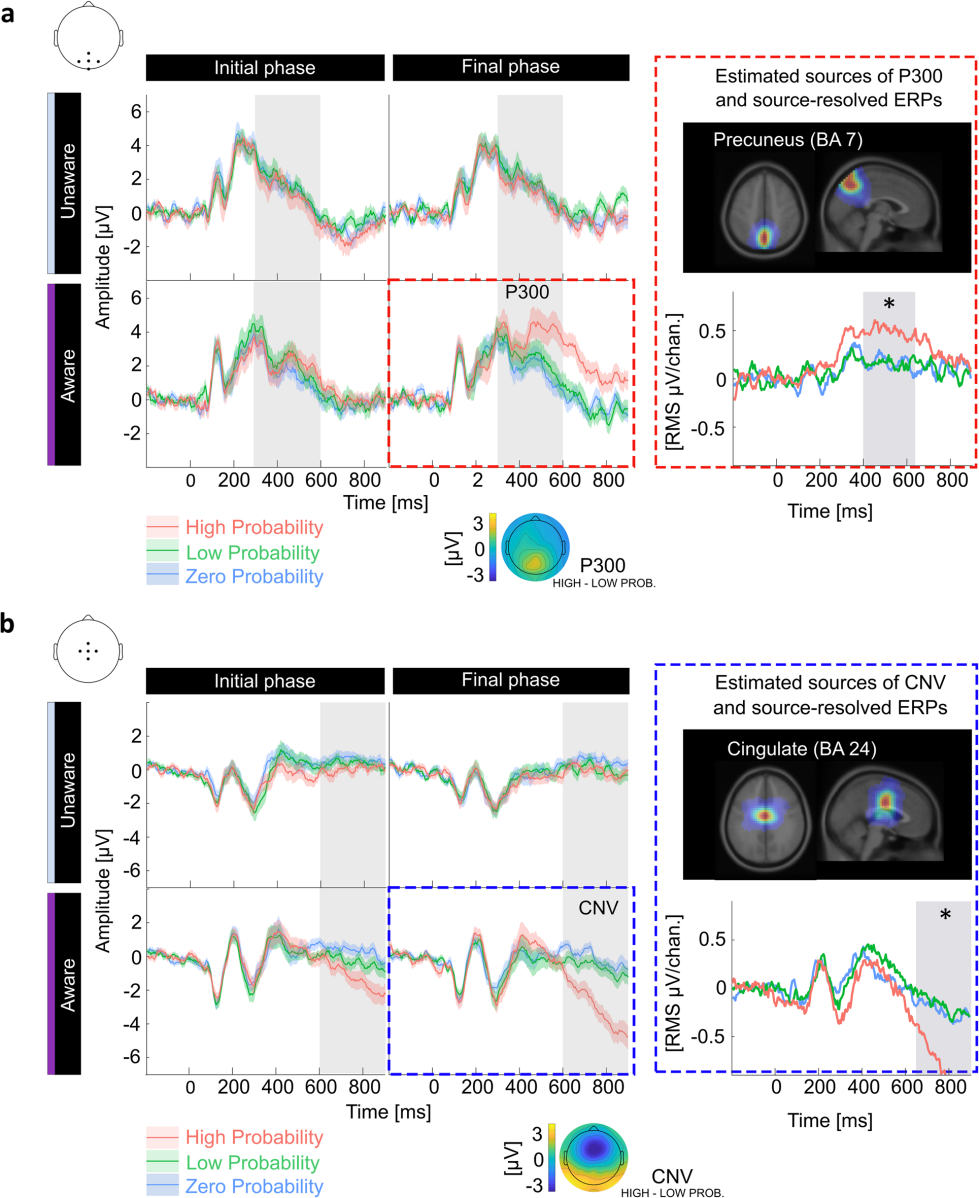
Only contingency-aware subjects showed contingency-related neural changes in cue processing. ERPs are shown in two spatiotemporal regions of interest (ROI) related to the cue-P300 (**a**) and the CNV (**b**). A grey background indicates the time window of these ROIs. ERP traces (group-level grand average and SEM) were plotted for each condition. Right-most panels present the main neural source of the respective ERPs. The location of the source within the brain is indicated by an equivalent-dipole density map overlaid on horizontal and sagittal planes of a brain image. The scalp-projected activity of these sources is shown beneath. Asterisks signify a significant difference between the High- and Low Probability conditions (nonparametric permutation test with Bonferroni-Holm correction).

Only in the contingency-aware group the P300 amplitudes elicited by High Probability cues increased over sequential trials, eventually becoming larger than amplitudes elicited by the other cues (see Fig. 2a and Supp. Fig. S1 for the estimated amplitude slopes over trials). This observation was corroborated by the LMM analyses (Supp. Tables S4–S6) and by ANOVA performed on the LMM-estimated P300 amplitudes (Table 2). As expected from prior studies^40,41^, the differential wave (High Probability P300 minus Low Probability P300) was pronounced at mid-parietal scalp locations and latencies of about 400–650 ms. It was estimated to originate primarily from the middle occipital gyrus (BA-19), precuneus (BA-7), and inferior occipital gyrus (BA-18). These three sources accounted for 73.5% of the signal variance. The differences in source-resolved ERPs observed between the High and Low Probability cues were significant only in the precuneus (t(21) = 3.17, *p*-value = 0.002; for remaining statistical results, see Supp. Table S14). The source activation was greater for the High Probability cues than for other cues, consistent with the potentials on the scalp (Fig. 2a). The contingency-unaware group showed no differentiation of cue-P300 amplitudes between conditions.

**Table 2:** 3-way ANOVA (type III sums of squares) on the LMM estimates of cue-P300 amplitudes.

	Sum Sq	Num. df	Den. df	*F*	*p*
Condition	32.71	2	412.4	0.72	0.485
Trial	428.88	1	6795.7	19.00	< 0.001
Group	11.85	1	59.0	0.53	0.472
Condition:Trial	85.09	2	6799.0	1.88	0.152
Condition:Group	17.72	2	412.4	0.39	0.676
Trial:Group	196.60	1	6795.7	8.71	0.003
Condition:Trial:Group	241.89	2	6799.0	5.36	0.005

Independent variables as in the RT model.

The CNV potential was also found only in the contingency-aware group. The CNV amplitudes elicited by highly predictive cues increased gradually, eventually becoming significantly more negative than for the other cues (Fig. 2b and Supp. Fig. S2 for the estimated amplitude slopes over trials). This was corroborated by the LMM analyses (Supp. Tables S7–S9) and by ANOVA performed on the LMM-estimated CNV amplitudes (Table 3). The signal in the CNV-dominated time window (650–900 ms) was attributed to the activity in the anterior cingulate (BA-24), inferior occipital gyrus (BA-18), and precentral gyrus (BA-6). Together, they accounted for 76.6% of the total signal variance. The differences in source-resolved ERPs between the High and Low Probability conditions was significant only in the anterior cingulate (t(30) = − 3.03, *p*-value = 0.002; for remaining statistical results see Supp. Table S14). The deflection of the source-resolved ERP was more negative for the High than for the Low Probability cues, reminiscent of the scalp-level CNV.

**Table 3:** 3-way ANOVA (type III sums of squares) on the LMM estimates of CNV amplitudes.

	Sum Sq	Num. df	Den. df	*F*	*p*
Condition	115.10	2	264.7	3.86	0.022
Trial	543.60	1	6790.8	36.45	< 0.001
Group	36.46	1	77.6	2.44	0.122
Condition:Trial	153.48	2	6795.2	5.15	0.006
Condition:Group	18.91	2	264.7	0.63	0.531
Trial:Group	277.14	1	6790.8	18.58	0.000
Condition:Trial:Group	167.97	2	6795.2	5.63	0.004

Independent variables as in the RT and cue-P300 model.

In sum, learning influenced only the ERPs elicited by the highly predictive cues. The observed ERP pattern resulted from the precuneus activation, tightly followed by the anterior cingulate cortex activation. Importantly, this effect was present only in subjects who became aware of the contingencies.

### Both cue-elicited ERP effects are linked to behavioral adaptation

**Figure 3:**
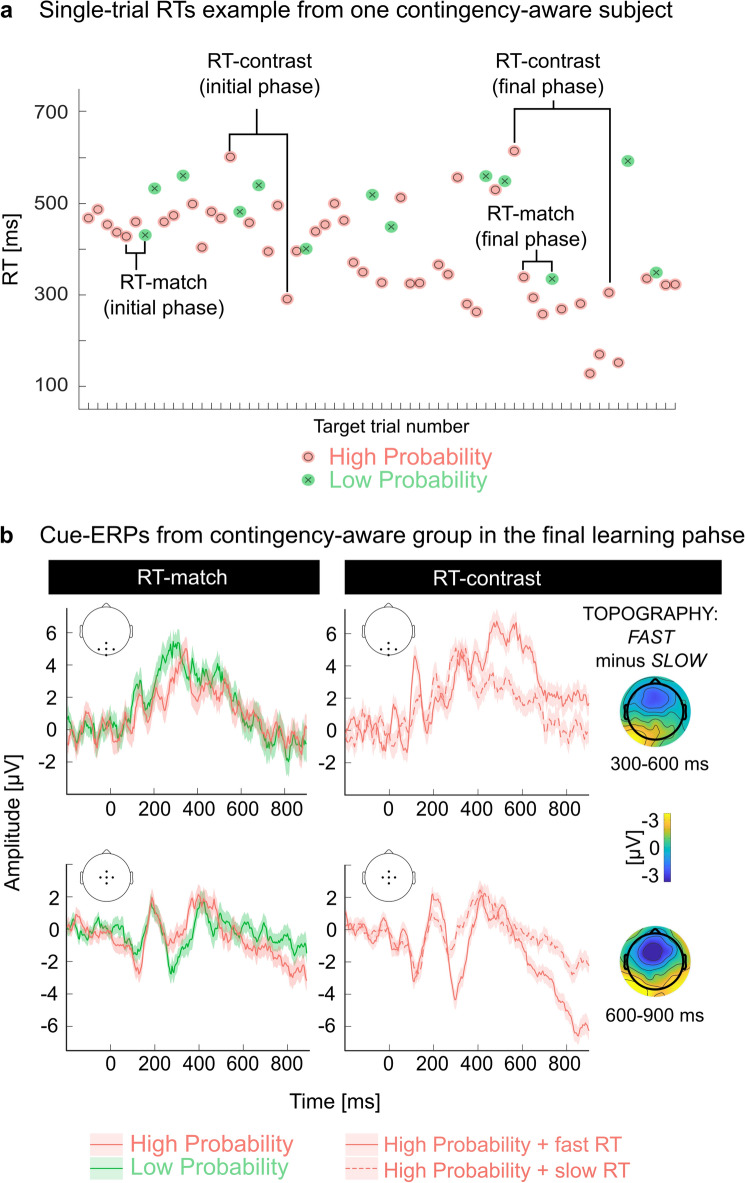
Both cue-evoked ERPs are related to behavioral optimization. (**a**) Visual explanation of the RT-contrasting and RT-matching procedure using Individual subject RTs. (**b**) Cue-ERPs from the final phase and contingency-aware subjects after RT-matching (left-hand side) and RT-contrasting (right-hand side). ERPs are plotted (grand average and SEM) within the previously used regions of interest for P300 (top row) and CNV (bottom row).

Next, we analyzed the trial-wise relationship between the event-related brain potentials (cue-P300 and CNV) and the acceleration of responses during learning in the contingency-aware group. In this group, all learning-related changes co-occurred, leaving the question of whether both cue-ERPs are functionally related to acceleration. We asked whether enhanced cue-P300 and CNV were only associated with trials showing the behavioral effect. An inherent variability of individual RTs that exists even in the final phase of the learning task allowed us to test two predictions:

1. If the ERPs reflect contingencies (or contingency awareness) independently of the behavior, they should be present in contingency-aware subjects at all times in the late learning phase, i.e. also when comparing High with Low Probability trials associated with similar RTs (see methods: RT-matching procedure). We found that RT matching (within ± ΔRT, for details see Materials and methods) eliminated the enhancement of cue-P300 and CNV (previously found in contingency-aware group); the RT-matched ERPs showed no difference between conditions for all tested ΔRT values (cluster-mass permutation test; the cluster-related *p*-values ≥ 0.105, 0.422, and 0.475 for ΔRT = 5, 10 and 15, respectively, see also Supp. Table S20). Within our regions of interest, the ERPs obtained for RT-matched trials show a great deal of overlap (Fig. 3a). This suggests that the neural correlates of learning are determined by behavior, rather than by contingency (Δp) or contingency awareness alone.
2. Furthermore, if the ERPs reflect contingencies (or contingency awareness) independently of the behavior, they should be absent while comparing trials belonging to the same condition (High Probability), but associated with different response times (see methods: the RT-contrasting procedure). Instead, we found a significant difference in cue-ERP amplitudes (cluster-mass permutation test, all cluster-related *p*-values < 0.01) between High Probability trials associated with fast and slow RTs. This effect was present in contingency-aware subjects in both task phases, but not in the unaware subjects (Supp. Figs. S9–S10). The resultant ERP pattern within our regions of interest bore a striking similarity to the original cue-P300 and CNV (Fig. 3b; compare with Fig. 2a,b).

Together, these results demonstrate that both the enhancement of cue-P300 and the CNV are functionally related to anticipatory motor preparation that markedly reduces the RTs.

### SL_C_ differently affects target processing in contingency aware and unaware subjects

Last, we explored the effects of statistical learning on target processing and their dependence on contingency awareness. By examining the P300 response, we compared how the two groups responded to the predictable targets in the oddball-SL task versus randomly occurring targets from the oddball-control task. The analyses show learning-related changes over time and in specific brain regions.

**Figure 4:**
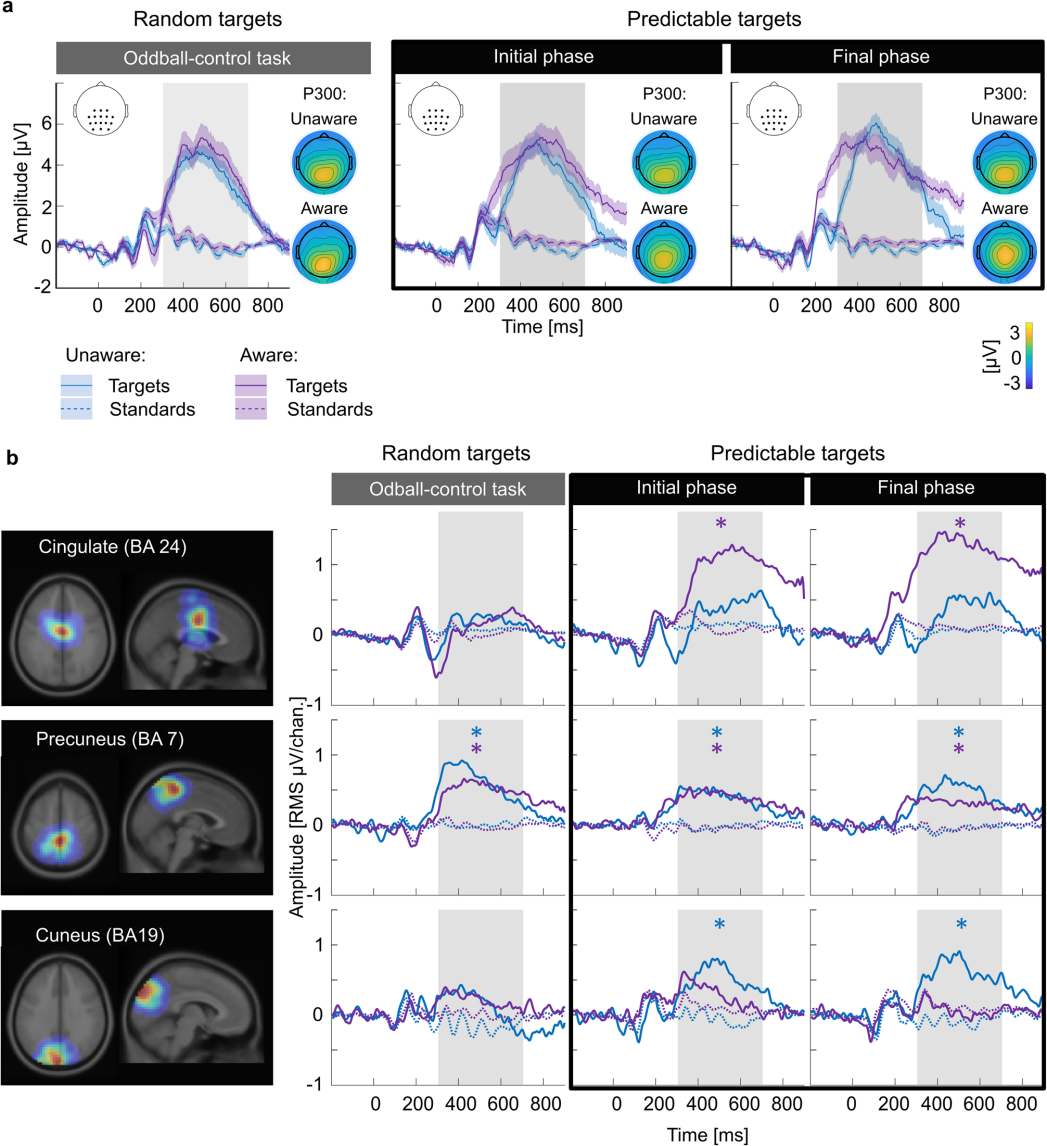
Statistical learning of contingencies influences the relative contributions of brain sources to target-evoked P300 potential. (**a**) ERPs (group average and SEM) elicited by standards and targets within the target-P300 ROI during the oddball-control task and two phases of the oddball-SL task. (**b**) Sources determined as the main generators of the target-P300; left-hand side: dipole density maps in horizontal and sagittal brain planes; right-hand side: source-resolved ERPs for standard and target trials. The presence of a source-resolved P300 component was examined (nonparametric permutation test; asterisks mark significant target vs. standard difference after Bonferroni-Holm correction).

The target-evoked P300 was present in both groups (Fig. 4a); we found no between-group differences with regard to the amplitudes elicited by random targets from the oddball-control task (cluster-mass permutation test; all *p*-values ≥ 0.399), standards (all *p*-values ≥ 0.416), or the differences between these two conditions (all *p*-values ≥ 0.484).

In the learning task, we focused on the most numerous, highly predictable targets (i.e., those occurring in the High Probability condition). The target-P300, defined as the amplitude difference between target and standard trials, became larger over time due to increased amplitudes elicited by targets over subsequent trials (Table 4; see also Supp. Fig. S3). The group factor did not significantly improve the LMM fit (Supp. Tables S10–S11); hence, contingency awareness did not markedly influence the scalp-recorded target-P300 amplitudes.

**Table 4:** 3-way ANOVA (type III sums of squares) on the LMM estimates of the target-P300 potential.

	Sum Sq	Num. df	Den. df	*F*	*p*
Stimulus	1005.47	1	72.0	111.97	< 0.001
Trial	50.90	1	8698.2	5.67	0.017
Stimulus:Trial	100.68	1	8698.2	11.21	0.001

Stimulus (targets/standards in High Probability condition), Trial—continuous variable based on event latencies.

On the contrary, the neural sources of the P300 were influenced by contingency awareness. In the contingency-unaware group, the primary sources of P300 in the control task were the cuneus (BA-19), anterior cingulate (BA-24), and superior temporal gyrus (BA-42), which together accounted for 50.9% of the P300-related signal variance. In the learning task, the primary sources of P300 were the cuneus (BA-19), precuneus (BA-7), and posterior cingulate cortex (BA-23). These three sources together accounted for 71.7% and 79.4% of the P300-related signal variance in the initial and final phases, respectively.

In the contingency-aware group, in both tasks the primary sources of P300 were anterior cingulate (BA-24), precuneus (BA-7), and cuneus (BA-19). They accounted for 68.6%, 81.9%, and 82.6% of the P300-related signal variance in the control task, in initial, and final oddball-SL task phases, respectively.

The source-resolved activity attributed to precuneus showed a significant P300 component in both groups, tasks, and oddball-SL task phases (permutation tests; all *p*-values < 0.001; Fig. 4b). During the oddball-SL task, the ‘aware’ group additionally showed source-resolved P300 in the cingulate cortex (both phases’ *p*-value < 0.001); By contrast, contingencies influenced the ‘unaware’ subjects’ EEG by inducing an additional P300 component in the cuneus (both phases’ *p*-value < 0.001; remaining statistical results in Supp. Table S17).

In summary, precuneus activity contributed to the target-elicited responses independently of predictability and contingency awareness. The cingulate cortex and cuneus, on the other hand, were differentially activated, depending on contingency awareness.

## Discussion

This study used two visual oddball tasks requiring speeded responses to target stimuli. Unbeknown to the participants, the stimuli sequence in one of the tasks included three types of target cues, allowing for statistical learning of cue-target contingencies (SL_C_) and, consequently, utilizing them proactively to speed up responses. The order of stimuli in the other (control) task was random. Our central finding is that SL_C_ led to proactive behavior solely in subjects who gained explicit knowledge about those contingencies (contingency-aware). The contingency-unaware group did not optimize behavior during task performance, which suggests that contingency awareness is necessary for proactivity based on cue-triggered anticipation. Nonetheless, even in the absence of contingency awareness, we observed an alteration of brain responses to predictable relative to random targets and a better-than-chance group performance in a post-exposure forced-choice test. These two effects found in the unaware group are interpreted as implicit SL_C_. Together, the results document that despite identical experimental conditions, SL_C_ may lead to different adaptations and that the scope of adaptations unlocked by implicit learning is limited relative to those accompanied by contingency awareness. The two qualitatively different outcomes of SL_C_ are discussed below.

Only the contingency-aware group shortened their RTs selectively to the predictable targets, evidencing SL_C_. Learning was also evidenced by selective modulation of ERP responses to High Probability cues. Importantly, since all types of cues occurred equally often, the differentiation of cue-ERPs across conditions could not be attributed to occurrence frequencies^34,47,48^. Note also that the neural measures failed to differentiate between Zero- and Low Probability cues, showing better alignment with the distinction between positive (Δp > 0) and negative (Δp < 0) contingencies than with the distinction based on target probabilities^49,50^. The ERP effects consisted of the contingent negative variation (CNV) and an enhancement of the cue-P300 in the High Probability condition. The presence of the CNV component indicates the role of anticipatory motor preparation^13,15,33^. The group-specificity of both behavioral and neural effects suggests that proactive exploitation of cue-target contingencies is only possible with contingency awareness.

At first, this conclusion may seem controversial given the well-documented preponderance and diversity of implicit statistical learning^6,51^. However, as stated in the introduction, most paradigms assess statistical learning by applying post-exposure measures and do not address the specific question of whether proactivity is achievable without contingency awareness. As pointed out by Dale et al.^26^, many claims about implicit anticipation during statistical learning tasks are ungrounded due to methodological issues (see also^52^), while sound demonstrations of anticipatory and predictive processing tend to correlate with explicit knowledge about the statistical regularities^26,27^. An implicit shortening of response times often attributed to anticipation is reliably observed in motor sequence learning paradigms. Unlike contingency learning explored in our study, those adaptations are rooted in procedural learning and persistent re-enactment of the regularities by the motor system. They involve a distinct neural circuity with a prominent role of the cerebellum^53^ and may be less dependent on explicit knowledge than contingency learning^54^. The relation between implicit or unconscious processes and proactive cognitive control is an active and hotly debated research topic^24,55–57^. Several EEG studies claim to show signatures of anticipatory neural modulation—the CNV potential—due to implicit proactive control^36,38^. Crucially, these studies explored the implicit acquisition of sequence *timing*, particularly the duration of inter-stimulus intervals^37^. The anticipation of stimulus onset time may be qualitatively different from the anticipation of stimulus identity. During task performance, the distribution of inter-stimulus intervals translates into the distribution of intervals between sensory and motor responses, making a case for sensorimotor learning. By applying randomly jittered inter-stimulus intervals, we focused solely on identity-based anticipation that likely involves distinct neural mechanisms. Finally, the relation between SL_C_ and contingency awareness may also depend on other stimulation parameters. As established by the long tradition of human conditioning research^1,58–61^, when a temporal gap separates conditioned and unconditioned stimuli (equivalents of cue and target), behavioral adaptation requires contingency awareness and depends on hippocampal integrity^59,62–64^. Implicit adaptations, on the other hand, are hippocampus-independent but require a partial overlap of the stimuli^55,59,65^. Since our stimuli were temporally separated, the discussed finding is consistent with this line of research (see also^18^).

Careful examination of the single-trial relationship between behavioral and neural indices of SL_C_ revealed that both cue-ERP effects occurred only on trials followed by relatively fast button presses. Hence both the CNV and the enhanced cue-P300 were linked to the behavioral adaptation and not the acquisition of knowledge per se. While the CNV is widely associated with anticipation and preparation –processes that speed up motor responses^12,13,33,43,44^—the functional role of the enhanced cue-P300 in the context of SL_C_ has never been firmly established^4,41,42^. It has been suggested that it reflects contingency awareness and may occur independently from the behavioral indices of learning^4,40^. If so, the EEG measures could track SL_C_ regardless of whether the subject is able or willing to follow task instructions. On the contrary, our single-trial analyses demonstrated that the cue-P300 effect was present only when the High Probability cues were followed by relatively fast motor responses. These fast, anticipatory responses were mostly absent in the initial task phase but ubiquitous in the final phase. Yet, even in that final phase, the individual RTs were highly variable. The presence of relatively slow RTs occasionally occurring between the fast ones suggests that, at this stage, subjects already gained the ability to behave proactively but did not apply it in some trials—perhaps due to a temporary lapse of attention or decreased motivation. The absence of the cue-P300 effect in those trials strongly suggests that cue-P300 enhancement—similarly to CNV—relates to proactivity.

Given the interpretation, our source analysis of the two cue-ERPs elucidates the neural substrates underpinning two stages of proactive behavior. A two-stage process is consistent with a computational model proposed by Lieder and Iwama^11^, consisting of a reactive recall of the cue's relevance to the task goal (stage 1) and proactive preparation for the target-related action (stage 2). The cue-P300 and the CNV effects presumably reflect those reactive and proactive processing stages. Supporting this claim, the modulation of the cue-P300 originated mainly from precuneus—a region within the parietal cortex involved in a cued recall of learned associations^66–68^, particularly associations formed by sudden comprehension of abstract relations^69^. The parietal cortex has already been implicated in reactive aspects of cognitive control as the anatomical structure supporting associations-mediated reactivation of task goals^10,70^. Interestingly, also targets consistently evoked precuneal activity regardless of the contingencies or contingency awareness. Therefore, as a result of learning, the neural responses to cues seem to acquire some characteristics of target-related responses, which may reflect the cue-induced reactivation of target representation. This reactivation seems functionally relevant to the subsequent proactive preparation for targets, as corroborated by functional connectivity between the precuneus and a supplementary motor area facilitating fast responses in learning tasks^71^. The CNV signifying anticipatory preparation for targets originated mainly from the posterior part of the anterior cingulate cortex (ACC), also referred to as the mid-cingulate cortex^72^. This brain area is thought to implement motor control by 'translating abstract cognition and intentions to actions’^73,74^.

Learning also influenced the target-evoked responses of the contingency-aware group: the predictable targets elicited larger ACC responses than randomly occurring targets did. This activity could not be directly related to the RT speed-up as it was mainly observed at latencies exceeding the average RT. More likely, the ACC activity reflects updating the mental model of cue-target contingencies or evaluating the target as an outcome of learning-induced expectations, which provides the basis for choosing an optimal behavioral strategy^47,75–77^. While ACC has most widely been associated with prediction error signals^76,78^, signaling confirmed predictions is also important as a complementary side of the cognitive cycle that enables behavioral optimization on a trial-to-trial basis^79^.

Although the data emphasize the role of the precuneus and the ACC in the proactive exploitation of contingencies, additional brain structures also showed learning-related activity, albeit less robust (see Supp. Table S13). Despite the converging evidence from prior studies showing hippocampal involvement in contingency-aware subjects^39,45,46^, and its relevance to statistical learning in general^1,80^, our analyses did not reveal this source. Given the limited sensitivity of EEG to detect deep brain sources, this absence is not surprising. The hippocampus, however, forms connections with the precuneus, and their functional connectivity is known to increase during the presentation of High Probability cues^81^. Therefore, the precuneal-cingular network involved in SL_C_-induced proactivity is likely to receive contributions from the hippocampus.

The contingency-unaware subjects did not show online behavioral adaptation to statistical regularities: their RTs to predictable targets did not decrease. Consistent with the discussed relatedness of cue-elicited ERPs to anticipatory behavior, this group's neural responses to cues also remained unchanged. The combined results of the questionnaire, response time measures, and cue-evoked brain potentials suggest that subjects lacking contingency awareness were unable to proactively use the target cues to facilitate behavior. The above measures alone provide no evidence of learning. In particular, they do not demonstrate implicit statistical learning. However, expanding on previous studies that used this paradigm but did not analyze target-related responses^40,41^, we found that exposure to contingencies affects neural responses to predictable targets: the activity in visual association areas became larger for predictable than for randomly occurring targets. A similar result—i.e., greater activations within visual association areas for predictable than for random visual sequences without regularity awareness—has been reported in an fMRI study^82^. Although traditionally, any claim about *learning* would require behavioral evidence, changes in brain activity that reflect environmental regularities have already been established as a measure of implicit statistical learning, even when accompanied by only weak or no behavioral adaptations^82–84^. Furthermore, when neural measures are not available, it is common to assess implicit statistical learning based on post-exposure memory tests, e.g., completion tasks, familiarity judgments, and 2AFC procedures^2,51,85^. Indeed, we found that despite the lack of proactivity, the unaware group scored above chance in a post-exposure alternative-choice test, i.e., when prompted to guess which stimulus usually preceded the target (note that this was after the subjects claimed complete randomness of the sequence). Together, the modulation of target-evoked brain responses within visual association areas and the implicit bias in an alternative-choice test can be taken as an indication of implicit SL_C_. Importantly though, this implicit SL_C_ appears insufficient for a proactive exploitation of contingencies. Hence, instead of assessing the ability to learn contingencies in zero–one terms, our study provides a deeper understanding of the functional limits and qualitative differences between implicit and explicit modes of SL_C_^18^.

What neural processes lead to these differential outcomes and what factors influence the learning mode still need to be clarified. After all, both groups were exposed to the same conditions. Implicit acquisition of statistical regularities is often explained by associative theories of learning, which entail an incremental strengthening of associations between co-occurring stimuli and stimulus–response pairs^58,60^. In neurophysiological terms, these processes relate to Hebbian learning mechanisms; they are largely automatic and require repeated exposure over a prolonged time. Consistent with the notion that statistical learning takes time^4,86^, our 15 min long task might have induced associative processes in the contingency-unaware group but captured only their initial phase. Alternative theories on human contingency learning postulate inferential reasoning as the driving force of learning^60,87^. Inferential reasoning, consisting in formulating and validating hypotheses, is a cognitively controlled process that leads to explicit knowledge about contingencies^88–90^, possibly through a sudden insight^69,86^. In contrast to associative processes, it may occur fast, taking only a few trials to form the initial hypothesis. The debate between proponents of associative and inferential theories is ongoing^89,91^. The dissociation of outcomes in our study suggests that inferential processes might have been the driving force of learning in the contingency-aware group. In contrast, associative processes might have contributed to the outcomes of the contingency-unaware group.

A final comment relates to the outcome of our supplementary classification of individual subject RTs. A general advantage of gaining contingency awareness is the flexibility of the acquired behavior^18^. Contingency awareness offers the possibility to use a proactive strategy, and not all of our subjects decided to use it. Note that despite applying the speed pressure, the explicit goal of the oddball tasks was to respond to targets and refrain from responding to other stimuli. During the questionnaire, a few contingency-aware subjects spontaneously reported that they ignored the High Probability cues and focused solely on targets because they realized that the predictive relation is not 100% reliable. These subjects seem to consciously refrain from the proactive strategy due to a strong bias toward accuracy in situations involving speed-accuracy tradeoff.

In conclusion, our behavioral and electrophysiological results contribute to the debate on the mechanisms of statistical learning about cue-target contingencies and suggest functional limits of implicit SL_C_. Although neural activity within the visual cortex and post-exposure behavioral measures demonstrated implicit learning, these presumably associative processes were insufficient to drive proactive behavior. Proactive utilization of cue-target contingencies entails anticipatory motor preparation triggered by specific cues and involves brain regions related to cognitive control. Importantly, this behavior seems to be non-automatic, flexible, and enabled by contingency awareness.

## Materials and methods

### Participants

Forty-eight adults (age: 24.4 ± 4.8 (M ± SD), 54% female, 100% right-handed) participated in the study. All signed an informed consent and received 20€ remuneration. The experimental procedure was approved by the ethics committee of the Hannover Medical School, and all methods were performed in accordance with the Declaration of Helsinki.

### Tasks and stimuli

During one EEG session, participants performed two oddball tasks (oddball-SL and oddball-control; see below) under speed pressure. In the beginning, subjects were presented with five visual stimuli, from which one was indicated as a target. The subjects were instructed to attend to a sequence of these stimuli and to press a button every time they saw the target. After every block of trials, subjects were given feedback on their average response time (RT) and encouraged to respond faster. Blocks were separated by a break of minimum 30 s. Two task versions (with colors and with shapes, see Fig. 1a) were used, so that each subject could perform one task (e.g., oddball-SL task) with color stimuli and one (e.g., oddball-control) with shape stimuli. The assignment of task versions and the task order were balanced across participants (see Supp. Table S22 for individual subject information including the assignment). Since our preliminary analyses did not reveal influence of task version on the emergence of contingency awareness (Supp. Fig. S4), on RT-based measures of SL_C_ (Supp. Fig. S5), or on the ERP indices of contingency learning (Supp. Tables S4 and S7; Supp. Fig. S6), the data from both task versions were combined. The categories assigned to individual stimuli included *standards*, *targets*, and three *deviants*. Their occurrence frequencies (expressed as a percentage of the entire sequence) were: 68.6% (standards), 8.6% (targets), and 7.6% (each deviant). The assignment of stimulus categories to specific colors or shapes was randomized for each participant.

*The oddball-SL task* was modeled on Jost’s paradigm^41^. The stimulus sequence was probabilistic, i.e., the occurrence of targets was governed by contingencies and therefore allowed for statistical learning. The sequence consisted of a continuous succession of short stimuli series. Every series began with 1–5 standards followed by one deviant—here functioning as a *cue*. Each cue was associated with a different probability of being followed by a target (‘transitional probability’, TP), providing the basis for three within-task conditions: High-, Low-, and Zero Probability (Fig. 1b). The transitions between series were inconspicuous. There were seven blocks, each consisting of 24 series (8 per cue). Both the TPs and TP-derived contingency values (Δp) had been widely used to describe predictive relations, although different research fields have a proclivity to use either one of them. Here, we report both. The contingency value for the High Probability cue was determined as the probability of it being followed by a target (7 out of 8 times in a block) minus the probability of the target appearing without that cue^49,50^ (i.e., twice per block for the Low Probability and never for the Zero Probability cue): $\Delta {p}_{HP}= \frac{7}{8}- \frac{2}{16}=0.75.$ Analogous calculations for the Zero- and Low Probability cues yield Δp =  − 0.19 and Δp = − 0.56, respectively. Initial and final phases were defined as Blocks 1–3, and 5–7, respectively. Directly after this task, subjects were surveyed to determine whether they became aware of the contingencies. Subjects were classified as having explicit knowledge (contingency-aware) if they reported the predictive relation between a High Probability cue and the target. Subjects who were unable to report any regularity and claimed that the sequence was random (contingency-unaware) were asked to guess which stimulus usually preceded the target (see Supp. Fig. S7).

*The oddball-control task* did not include contingencies. It served to obtain measures of a typical P300 component^47^, i.e., elicited by rare and randomly occurring targets. It was identical to the oddball-SL task except: (1) the stimuli were presented in a randomized order while avoiding immediate target repetitions, and (2) the task was limited to 3 blocks (equal to one phase of the oddball-SL task).

The stimuli were generated using Psychophysics Toolbox PTB-3 (version 3.0.14^92^) running under Matlab (version 9.6.0, R2019a) and displayed on a 1920 × 1200 pixel View Pixx LCD monitor (VPixx Technologies Inc.) with a 120 Hz refresh rate. Subjects were seated at a monitor-to-forehead distance of about 1.35 m. Stimulus size spanned 2 degrees of visual angles. The stimuli were displayed for 200 ms, with a varying inter-stimulus interval of 800–1400 ms.

### EEG data acquisition and processing

Electroencephalographic signals (EEG) were recorded using BrainAmp MR + amplifiers (Brain Products, Munich, Germany) and 64 active Ag/AgCl electrodes mounted in standard, international 10/10 system caps (EASYCAP GmbH, Germany). Impedances were kept below 15 kΩ. The analog signal was filtered online with a low cutoff frequency of 0.016 Hz, a high cutoff frequency of 250 Hz, and digitized at a sampling rate of 1000 Hz.

EEG signal preprocessing was performed using EEGLAB toolbox^93^ and custom-written Matlab scripts. The following preprocessing steps were performed on individual datasets (2 datasets per subject, one per task). Data were down-sampled to 250 Hz and referenced to the average of all channels by applying a multi-stage robust referencing scheme. This scheme (‘PREP pipeline’) minimized the influence of noisy channels (see^94^ for details). An average of 0.5 (SD = 0.7) channels per dataset was identified as noisy. These channels and the original online reference (FCz) were later interpolated.

Independent component analysis (ICA) was performed to separate brain activity from EEG artifacts^95,96^: ICA weights were trained on a portion of the signals that were high-pass filtered (0.5 Hz cutoff frequency, zero-phase Hamming-windowed sinc FIR filter), segmented (1 s segment duration), and automatically cleaned (EEGLab joint probability method, threshold = 2.5 SD). The ICA results were applied to data high-pass filtered with 0.15 Hz cutoff frequency (FIR filter as above). Independent components (ICs) classified with more than 90% probability as artefactual or less than 5% probability as brain-related were automatically rejected, and those classified with more than 90% probability as brain-related—automatically retained (EEGLAB plugin ICLabel ver. 1.2^97^). The decision about the remaining components was based on visual inspection. Recordings were epoched from − 200 to 900 ms relative to stimulus onset and baseline-corrected (baseline from − 200 to 0 ms). Target trials lacking a button-press response within 1000 ms (misses) and non-target trials followed by a button-press response (false alarms) were excluded. Finally, an average of 13.1% (SD = 4.1%) epochs per dataset was rejected based on EEGLAB data cleaning routines (joint probability criterion: 4 SD; abnormal trend detection: max slope of 50 μV per epoch; visual inspection).

### Behavioral data analysis

In every dataset, we calculated a hit- and a false-alarm score. Hits were defined as target trials on which a button-press response was given within 1000 ms after the target presentation. The hit score was the percentage of hits within all target trials. By analogy, the false-alarm score was the percentage of false alarms within all non-target trials. Due to ceiling accuracy, the sensitivity index (dʹ) was not determined. The response time (RT) was defined for hits as the latency between the target presentation and the subsequent button-press response.

### ERP analyses

We focused on two cue-related ERPs: (1) a modulation of P300^41^, and a contingent negative variation (CNV^13^). These ERPs have different latency and distribution over the scalp. To obtain the measures of the cue-P300, the ERP amplitudes were averaged within a region of interest (ROI) based on Jost et al.^41^: 300–600 ms post-stimulus latencies at five parieto-occipital sites (Pz, POz, PO3, PO4, Oz). The ROI for the CNV included the final 300 ms of an epoch (600–900 ms post-cue latencies) and central sites (FCz, Cz, C1, C2, CPz).

We also investigated the influence of contingency learning on target-related P300. It is known that the P300 latency and topography may vary depending on experimental conditions^47^. To account for the specificity of our 5-stimulus oddball design and to determine an appropriate ROI, we performed a cluster-mass permutation test on *combined* group data from the control task. The P300 was prominent within 300–700 ms post-target latencies and at parieto-occipital sites (C1, Cz, C2, CP3, CP1, CPz, CP2, CP4, P3, P1, Pz, P2, P4, PO3, PO4, POz). This ROI was later applied to individual group data. The control task provided measures of P300 related to random targets, while the High Probability condition from the oddball-SL task provided measures of P300 related to predictable targets.

### Matching and contrasting cue-related ERPs by RTs

88% of the High Probability cues and 25% of the Low Probability cues were followed by a target. If subjects responded to these targets, the cues could be associated with a specific RT. We examined the relation between ERPs elicited by these cues and the associated RTs using two complementary approaches:

*The RT-matching procedure* yielded pairs of Low and High Probability cues associated with similar RTs (i.e., RT difference smaller than a specified ΔRT). The aim was to compare cue-related ERPs associated with different contingencies but similar behavior. Pairs were created within a given group and task phase (for an illustration of the concept and an example of RT-matched pairs, see Fig. 3a). To make sure that the outcomes are not specific to any a priori defined ΔRT value, the procedure was repeated three times (for ΔRT = 5, 10, and 15 ms). Priority was given to within-subject pairs. If multiple matching possibilities existed, trials in closer temporal proximity were selected to form a pair. After exhausting within-subject matching possibilities, matching was performed between different subjects of the same group. On average, 67% (SD = 15) of the resulting pairs were within-subject pairs. Since fewer Low- than High Probability cues were followed by targets (6 vs. 21 per subject within a task phase), the maximum number of possible pairs was limited by the number of Low Probability trials. This amounted to 132 and 156 for the ‘aware’ and ‘unaware’ subjects, respectively. Due to EEG data cleaning that limited the number of available pairs, and the requirements of the RT-matching procedure, the final number of trial pairs ranged—depending on task phase and ΔRT (‘aware’: 94–111; ‘unaware’: 14–127).

*The RT-contrasting procedure* yielded pairs of High Probability cue trials associated with distinct (i.e., ‘fast’ and ‘slow’) RTs. The aim was to compare cue-ERPs associated with the same contingency but different behavior. The procedure was performed within a given group and task phase. To define the ‘fast’ and ‘slow’ responses, the distribution of RTs within the respective data portion was divided into three equal parts (see Supp. Table S21, Fig. S8). ‘Fast’ RTs were those below the 33.3 percentile of the distribution, whereas ‘slow’ RTs were those above the 66.6 percentile. The pairs consisted of two High Probability trials—one associated with a slow RT and one with a fast RT. Again, priority was given to within-subject pairs. The excess of trials (occurring whenever ‘fast’ and ‘slow’ trials of a given subject were not equinumerous) were subsequently used to create between-subject pairs. On average, 68% (SD = 19) of these pairs were within-subject pairs. By design, 21 High Probability within each task phase were followed by a target. Summing across subjects, this amounted to 462 (‘aware’) and 546 (‘unaware’) High Probability trials. These trials were further split into three speed categories, so the number of potential pairs was reduced to one-third. Due to EEG data cleaning that limited the number of available pairs, the procedure yielded 48 (‘aware’, initial phase), 52 (‘aware’, final phase), 70 (‘unaware’, initial phase), and 68 (‘unaware’, final phase) RT-contrasted trial pairs.

### Source estimation

To perform EEG-based source estimation, we followed standard practices of the dipole modeling method^95,98^. Briefly, the technique used 3D coordinates of scalp electrodes and a head model (here: three-shell boundary element model of the MNI standard adult brain) to determine locations of equivalent current dipoles that best explain the scalp distribution of ICA-derived independent components (ICs). ‘Plausible’ dipoles (see below) were clustered based on their spatial locations. The activation of each IC was back-projected to the scalp. Summed IC projections originating from a given cluster were root-mean-squared across all channels, yielding source-resolved ERP traces. The coordinates of a cluster centroid were used to assign a brain region to each source. The set of all clusters is referred to as a ‘source model’.

We constructed two source models: MODEL 1—to investigate the brain sources of cue-evoked ERPs—therefore based on contingency-aware subjects’ data from the oddball-SL task (Supp. Table S12); MODEL 2—to investigate the influence of SL_C_ and contingency awareness on brain sources of the target-P300—therefore based on datasets from all subjects and both tasks (Supp. Table S15). Every IC was fitted with either a single dipole (EEGLAB plugin DIPFIT 2.3^99^), or bilateral dipole pair (fitTwoDipole plugin^100^). The quality of the fit was assessed by calculating residual variance as defined in^101^). Plausible dipoles, i.e., located within the brain volume and associated with residual variance smaller than 15% (single dipoles) or 35% (bilateral dipoles) were included in the model (i.e., 52% and 53% of all available ICs in the case of MODEL 1 and MODEL 2, respectively). The 3D coordinates of the dipoles were used to run a k-means clustering algorithm (number of clusters set to 16 as an average number of clusters per dataset; threshold for outliers = 3 SD).

The subsequent steps were performed on the source (i.e., cluster) level. Each source’s contribution to the relevant ERP component was assessed by calculating the ‘percent of variance accounted for’ (PVAF,^101^), which informs how much EEG signal variance across channels could be explained by a given source relative to the remaining sources. The signal was defined in MODEL 1 as the difference in a cue-evoked ERP between the High and Low Probability conditions. We used two temporal windows: 400–650 ms and 650–900 ms, as dominated by the cue-P300 and the CNV, respectively). In MODEL 2, the signal was defined as the difference between targets and standards in a 300–700 ms time-window (corresponding to the target-P300 time window). Since PVAF is determined for all available sources, regardless of their role in generating a given ERP, focusing on a few sources associated with the highest PVAF is a common practice^98,102^. Here, the three sources associated with the highest PVAF were defined as *primary sources*. We reported cumulative PVAF for the three primary sources (main manuscript) and PVAF for individual sources (Supp. Table S13, S16). Due to the non-additive nature of this measure, the cumulative PVAF is different (usually smaller) than the corresponding sum of the individual PVAFs.

### Statistics

Linear mixed-effects models were applied to single-trial data to analyze learning-related changes over time (i.e., RT and ERP data in the oddball-SL task). Nonparametric permutation-based approaches were used for the remaining exploratory analyses.

*Linear mixed-effects models (LMM)* are recommended for EEG analyzes due to the hierarchical organization inherent in the data^103^. Moreover, LMM fitted to single-trial data can reveal within-task changes over time, making them particularly useful for studying learning-related processes^104^. Here, this approach was used to analyze single-trial RTs and ERP measures from the oddball-SL task. The distribution of RTs was non-normal, showing a long upper-side tail. Consequently, log-transformed RTs were used. LMMs were fitted using R package lme4^105^. In all models 'subject' was the contextual grouping variable. All categorical variables (e.g. ‘group’, ’condition’) were dummy coded. The ‘unaware’ group was used as a reference level within ‘group’, and the Low Probability condition as a reference level within ‘condition’. All models used 'trial' as a fixed-effect continuous predictor, representing the task's temporal dimension. The ‘trial’ variable was scaled to have values between 0 and 1 (corresponding to the first and the last stimulus in a task, respectively).

Our research question was whether the responses (either RTs or ERPs) undergo differentiation between the contingency-related conditions over the experimental time and whether we observe group differences in this respect. Therefore our initial full models included ‘condition’, ‘trial’, and ‘group,’ as well as their interactions as fixed effects. The initial random effects were specified as random intercepts across subjects and random slopes for condition and trial over subject (R syntax: (condition*trial | subject)), but these models did not converge. The most complex models that did converge included random intercepts over subjects and random slopes for condition over subject (R syntax: (condition | subject)). Model parameters were determined with the maximum likelihood method, and Satterhweite approximation was applied to estimate degrees of freedom and to obtain *p*-values. The final model structure was decided by a step-wise reduction in model complexity, first for random effects and then for fixed effects^105^. We identified the most parsimonious model that did not produce a significant drop in the model goodness-of-fit as assessed by the likelihood ratio test (LRT). The optimal model’s plots of residuals showed no violations of linearity or homoskedasticity^106,107^. Whenever model residuals deviated from normality, the data was mildly trimmed (see^106^ and Supp. Information). This critically revised optimal model is referred to as the final model. After calculating degrees of freedom by Satterthweite’s approximation method, the final model was subjected to analysis of variance (ANOVA). For further details, see Supp. Information.

*A cluster-mass permutation test* was used as a data-driven approach to analyze ERP differences beyond the predefined ROIs^108^. These tests (as implemented in the Mass Univariate ERP Toolbox^109^) were applied to:

1. examine cue-ERP differences between High- and Low Probability conditions after RT matching (separately for each group and oddball-SL task phase);
2. examine cue-ERP differences within the High Probability condition after RT contrasting (separately for each group and oddball-SL task phase);
3. determine a region of interest for target-P300 (using combined group data from the oddball-control task)
4. examine between-group differences in the ERP amplitudes elicited in the oddball-control task.

As a recommended practice to reduce the number of t-tests^109^, data were down-sampled to 100 Hz and limited to 100–900 ms post-stimulus latencies. 10,000 permutations were used, and neighboring data points with t-value exceeding a threshold corresponding to an alpha level 0.01 were clustered. Summed t-values within each cluster constituted the cluster-level statistics. Clusters that fell within the extreme 1% of the surrogate distribution (two-tailed test) were considered to signify a difference between experimental conditions. Thus the weak family-wise error rate (FWER) was controlled at a 0.01 level.

### Supplementary Information

Supplementary Information.

## Data Availability

Data obtained from the statistical learning task (oddball-SL) and used in this article are available in the Open Science Framework repository: https://osf.io/mzxju/?view_only=2c9a3b96922a4a928a6cca8cca87f6a1.
