# Supplementary material for: The relation between implicit statistical learning and proactivity as revealed by EEG
Source: Sci Rep. 2023 Sep 22;13:15787. doi: 10.1038/s41598-023-42116-y (PMC10516964; doi:10.1038/s41598-023-42116-y)
Supplement: Supplementary file 1 — Supplementary Information. [file 41598_2023_42116_MOESM1_ESM.docx]

# Supplementary Information

Contents

LMM reporting 3

Table S1. RT model selection 3

Table S2. RT estimates 4

Table S3. RT slope over trials – contrast analysis 4

Table S4. Cue-P300 amplitude model selection 5

Figure S1. Cue-P300 amplitude slopes over trials – LMM estimates 6

Table S5. Cue-P300 amplitude estimates 6

Table S6. Cue-P300 amplitude slope over trials – contrast analysis 7

Table S7. CNV amplitude model selection 8

Figure S2. CNV amplitude slopes over trials – LMM estimates 8

Table S8. CNV amplitude estimates 9

Table S9. CNV amplitude slope over trials – contrast analysis 9

Table S10. Target-P300 amplitude model selection 10

Figure S3. Target-P300 amplitude slopes over trials for standards and targets 11

Table S11. Target-P300 amplitude estimates for standards and targets 11

Estimation of neural sources 12

Table S12. Independent component clusters – model 1 13

Table S13. Sources’ contributions to signal variance – model 1 14

Table S14. Statistical tests for ERPs elicited by model 1 primary sources 14

Table S15. Independent component clusters – model 2 15

Table S16. Sources’ contributions to signal variance – model 2 16

Table S17. Statistical tests for ERPs elicited by model 2 primary sources 17

Additional results 18

Figure S4. Contingency awareness results – split by task version and task order 18

Figure S5. Response times – split by task version 18

Figure S6. Cue-P300 – split by task version 19

Figure S7. CNV – split by task version 20

Table S18. Individual subject RT slopes – contrast analysis 21

Figure S8. Individual subject RT estimates and their classification 24

Table S19. Accuracy and RTs in oddball tasks 25

Table S20. Cluster *p*-values after RT-matching 26

Figure S9. Cue-ERPs after RT-contrasting 27

Figure S10. Raster plots showing the result of RT-contrasting (aware group) 28

Method details 29

Figure S11. Questionnaire 29

Table S21. Boundaries of fast and slow RTs 30

Figure S12. RT distributions with fast and slow responses highlighted 30

Table S22. Subjects’ demographics and task assignment 31

## LMM reporting

Response times in the oddball-SL task

A linear mixed model (LMM) was fitted to the log-transformed response times (RT) data from the oddball-SL task^1^. Mild a priori data trimming (2.5%) yielded a dataset consisting of 2888 observations. The initial model formulated in R syntax was: RT ~ group*condition*trial + (condition | subject). By step-wise reduction of the initial model (*step* function from the lmerTest package^2^, it was determined that (a) the optimal model includes random intercept and slopes condition over subjects and that (b) the optimal model includes the group*condition*trial interaction term (see Table S1). Thus, the final model was identical to the initial model. This outcome supports the presence of group differences in contingency learning. A satisfactory distribution of model residuals was achieved by removing data points associated with extreme residuals^1^. The final model was thus fitted with 2825 observations (95.4% of the original data points). RT estimates for combinations of different factors (Table S2) and slope contrast (Table S3) were obtained using *emmeans* package^3^.

### Table S1. RT model selection

| Model | Eliminated term | AIC | BIC | LL | Dev. | χ^2^ | df | *p* |
| --- | --- | --- | --- | --- | --- | --- | --- | --- |
| Initial | none | -1502.4 | -1430.7 | 763.2 | -1526.4 |  |  |  |
| Step 1 | Slope by CONDITION | -1370.3 | -1310.7 | 695.2 | -1390.3 | 136.01 | 2 | < 0.001 |
| Step 2 | CONDITION:TRIAL:GROUP | -1415.0 | -1355.3 | 717.5 | -1435.0 | 91.36 | 2 | <0.001 |
| Underscored terms were retained | | | | | | | | |

### Table S2. RT estimates

| **Predictors** | **Estimates**  **(CI)** | **std. Beta**  **(standardized CI)** |
| --- | --- | --- |
| (Intercept) | 5.85  (5.79 – 5.92) | 0.30  (0.08 – 0.52) |
| Low Probability condition | *Reference* |  |
| High Probability condition : Unaware group | -0.01  (-0.09 – 0.08) | 0.87  (0.60 – 1.15) |
| High Probability condition : Trial | -0.40  (-0.48 – -0.33) | -0.46  (-0.55 – -0.38) |
| High Probability Condition : Trial : Unaware group | 0.46  (0.36 – 0.55) | 0.53  (0.41 – 0.64) |
| High Probability Condition | -0.03  (-0.09 – 0.03) | -0.93  (-1.14 – -0.73) |
| Aware group | *Reference* |  |
| Unaware group | 0.09  (0.00 – 0.17) | 0.05  (-0.25 – 0.35) |
| Trial | 0.11  (0.05 – 0.18) | 0.13  (0.06 – 0.20) |
| Trial : Unaware group | -0.15  (-0.24 – -0.07) | -0.17  (-0.27 – -0.08) |
| ICC | 0.53 |  |
| Marginal R^2^ / Conditional R^2^ | 0.229 / 0.637 |  |
|  |  |  |

### Table S3. RT slope over trials – contrast analysis

| Group | Slopes | |  | Contrasts | df | *t*-score | *p-*value |
| --- | --- | --- | --- | --- | --- | --- | --- |
|  | **High Probability** | **Low Probability** |  |  |  |  |  |
| Aware | 0.113 | -0.291 |  | **Low-High Probability** | 2743.2 | -11.03 | < 0.001 |
| Unaware | -0.039 | 0.014 |  | **Low-High Probability** | 2735.6 | 1.62 | 0.106 |
| Degrees-of-freedom method: Satterthwaite, *p*-value adjustment method: Tukey | | | | | | | |

Cue-P300 amplitudes in the oddball-SL task

An LMM was fitted to single-trial ERP amplitudes measured within the P300 region of interest. Mild a priori data trimming (<1%) yielded a dataset of 6957 observations. The initial model formulated in R syntax was: P300 amplitude ~ group*condition*trial + version*condition*trial + (condition | subject) to have the potential of accounting for differential effect of learning between groups and between task versions. Step-wise reduction of the initial model revealed that the optimal model: (a) includes random intercept and slopes for condition over subjects, (b) does not include task version as a factor, and (c) includes the condition*trial*group interaction term (Table S4). Thus, the final model was: P300 amplitude ~ group*condition*trial + (condition | subject). This outcome demonstrates group differences in the P300 amplitudes between conditions. A satisfactory distribution of model residuals was achieved by further removing data points associated with extreme residuals^1^. The final model was thus fitted with 6913 observations (99.3% of the original dataset).

### Table S4. Cue-P300 amplitude model selection

| Model | Eliminated term | AIC | BIC | LL | Dev. | χ^2^ | df | *p* |  |
| --- | --- | --- | --- | --- | --- | --- | --- | --- | --- |
| Initial | none | 42185.6 | 42356.8 | -21067.8 | 42135.6 |  |  |  |  |
| Step 1 | Random slope by condition | 42191.4 | 42328.4 | -21075.7 | 42151.4 | 15.82 | 5 | 0.007 |  |
| Step 2 | Version: Condition:Trial | 42181.8 | 42332.4 | -21068.9 | 42137.8 | 2.19 | 3 | 0.534 |  |
| Step 3 | Version:Trial | 42178.9 | 42315.8 | -21069.4 | 42138.9 | 1.09 | 2 | 0.579 |  |
| Step 4 | Version: Condition | 42178.5 | 42315.4 | -21069.2 | 42138.5 | 0.68 | 2 | 0.713 |  |
| Step 5 | Version | 42176.9 | 42307.0 | -21069.5 | 42138.9 | 0.48 | 1 | 0.490 |  |
| Step 6 | Condition:Trial:Group | 42182.8 | 42299.3 | -21074.4 | 42148.8 | 9.90 | 2 | 0.007 |  |
| Underscored terms were retained | | | | | | | | | |
|  | | | | | | | | | |


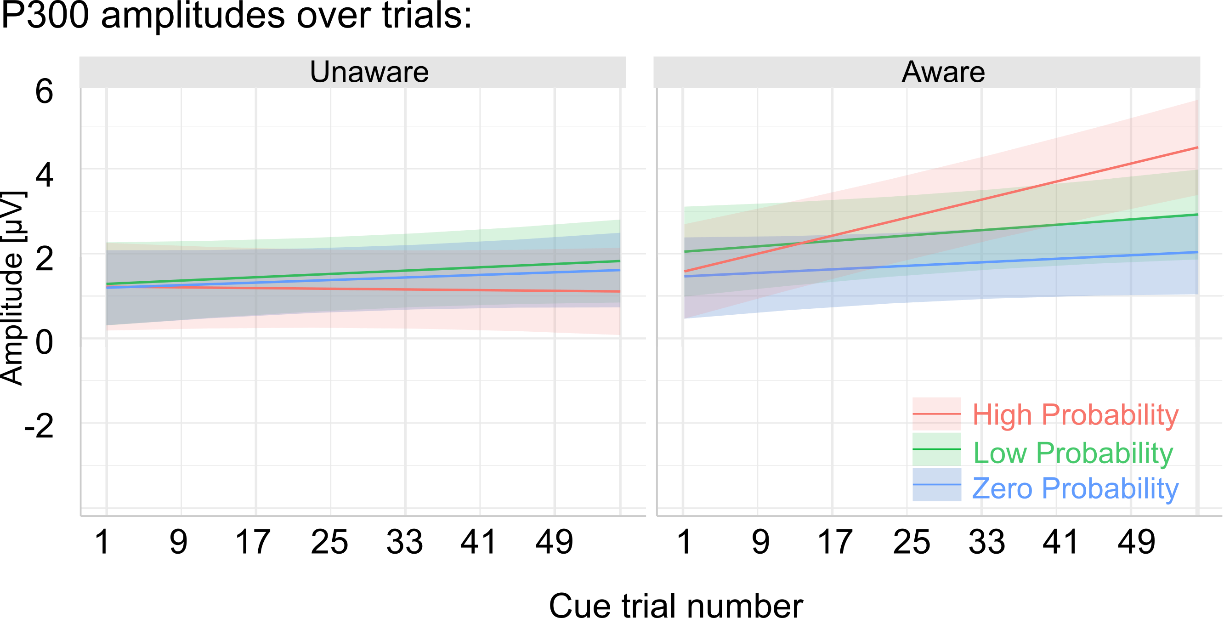


### Figure S1. Cue-P300 amplitude slopes over trials – LMM estimates

### Table S5. Cue-P300 amplitude estimates

| **Predictors** | **Estimates**  **(CI)** | **std. Beta**  **(standardized CI)** |
| --- | --- | --- |
| (Intercept) | 1.29  (0.31 – 2.27) | -0.06  (-0.22 – 0.11) |
| Low Probability condition | *Reference* |  |
| High Probability condition : Aware group | -0.40  (-1.60 – 0.80) | 0.18  (0.04 – 0.32) |
| High Probability condition : Trial | -0.65  (-1.96 – 0.66) | -0.04  (-0.11 – 0.04) |
| High Probability condition : Trial : Aware group | 2.70  (0.77 – 4.62) | 0.15  (0.04 – 0.25) |
| High Probability condition | -0.07  (-0.89 – 0.76) | -0.07  (-0.17 – 0.02) |
| Zero Probability condition | -0.09  (-0.87 – 0.69) | -0.03  (-0.11 – 0.05) |
| Zero Probability condition : Aware group | -0.49  (-1.63 – 0.65) | -0.11  (-0.23 – 0.01) |
| Zero Probability condition : Trial | -0.12  (-1.41 – 1.17) | -0.01  (-0.08 – 0.06) |
| Zero Probability condition: Trial : Aware group | -0.18  (-2.07 – 1.71) | -0.01  (-0.11 – 0.09) |
| Unaware group | *Reference* |  |
| Aware group | 0.76  (-0.68 – 2.20) | 0.18  (-0.07 – 0.42) |
| Trial | 0.54  (-0.38 – 1.45) | 0.03  (-0.02 – 0.08) |
| Trial : Aware group | 0.34  -1.01 – 1.68 | 0.02  (-0.06 – 0.09) |
| ICC | 0.16 |  |
| Marginal R^2^ / Conditional R^2^ | 0.02 / 0.18 |  |

### Table S6. Cue-P300 amplitude slope over trials – contrast analysis

| Group | Slope | | | Contrast | df | *t*-score | *p-*value |
| --- | --- | --- | --- | --- | --- | --- | --- |
|  | **High Probability** | **Low Probability** | **Zero**  **Probability** |  |  |  |  |
| Aware | 2.92 | 0.87 | 0.57 | **Low-High** | 6787.6 | -2.85 | 0.012 |
|  |  |  |  | **Low-Zero** | 6789.0 | 0.43 | 0.904 |
|  |  |  |  | **High-Zero** | 6792.9 | 3.28 | 0.003 |
| Unaware | -0.11 | 0.54 | 0.42 | **Low-High** | 6812.4 | 0.97 | 0.596 |
|  |  |  |  | **Low-Zero** | 6795.0 | 0.19 | 0.981 |
|  |  |  |  | **High-Zero** | 6812.7 | -0.79 | 0.707 |
| Degrees-of-freedom method: Satterthwaite, *p*-value adjustment method: Tukey | | | | | | | |

Cue-evoked CNV amplitudes

An LMM was fitted to single-trial ERP amplitudes measured within the CNV region of interest. Mild a priori data trimming (<1%) yielded a dataset consisting of 6953 observations. The initial model formulated in R syntax was: CNV amplitude ~ group*condition*trial + version*condition*trial + (condition | subject) to have the potential of accounting for differential effect of learning between groups and between task versions. Step-wise reduction of the initial model revealed that the optimal model: (a) includes random intercept and slopes for condition over subjects, (b) does not include task version as a factor, and (c) includes the condition*trial*group interaction term (Table S7). Thus, the final model was: CNV amplitude ~ group*condition*trial + (condition | subject), indicating group differences in the CNV measures related to contingency learning.
A satisfactory distribution of model residuals was achieved by further removing data points associated with extreme residuals. The final model was thus fitted with 6913 observations (99.3% of the original dataset).

### Table S7. CNV amplitude model selection

| Model | Eliminated term | AIC | BIC | LL | Dev. | χ^2^ | df | *p* |
| --- | --- | --- | --- | --- | --- | --- | --- | --- |
| Initial | none | 39245.2 | 39416.4 | -19597.6 | 39195.2 |  |  |  |
| Step 1 | Random slope by condition | 39285.2 | 39422.2 | -19622.6 | 39245.2 | 50.05 | 5 | <0.001 |
| Step 2 | Version: Condition:Trial | 39239.2 | 39389.8 | -19597.6 | 39195.2 | 0.01 | 3 | 0.999 |
| Step 3 | Version:Trial | 39239.8 | 39376.8 | -19599.9 | 39199.8 | 4.60 | 2 | 0.100 |
| Step 4 | Version: Condition | 39239.7 | 39376.6 | -19599.9 | 39199.7 | 4.49 | 2 | 0.106 |
| Step 5 | Version | 39238.0 | 39368.1 | -19600.0 | 39200.0 | 0.30 | 1 | 0.583 |
| Step 6 | Condition:Trial:Group | 39245.1 | 39361.5 | -19605.6 | 39211.1 | 11.12 | 2 | 0.004 |
| Underscored terms were retained | | | | | | | | |
|  | | | | | | | | |


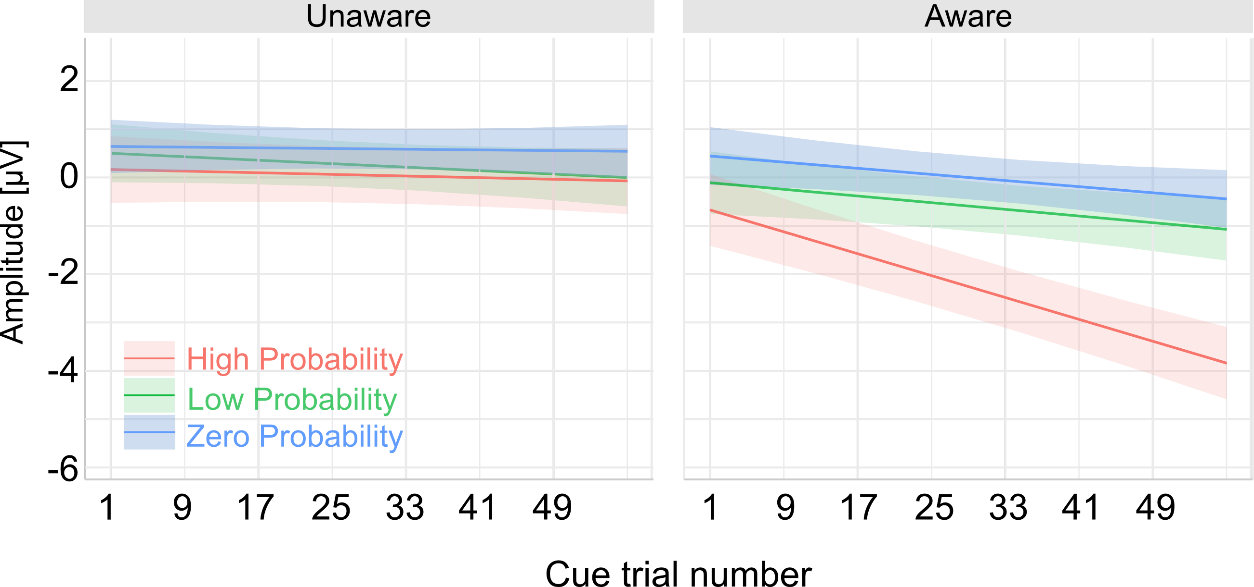


### Figure S2. CNV amplitude slopes over trials – LMM estimates

### Table S8. CNV amplitude estimates

| **Predictors** | **Estimates**  **(CI)** | **std. Beta**  **(standardized CI)** |
| --- | --- | --- |
| (Intercept) | 0.50  (-0.10 – 1.10) | 0.12  (0.01 – 0.23) |
| Low Probability condition | *Reference* |  |
| High Probability condition : Aware group | -0.23  (-1.20 – 0.74) | -0.35  (-0.49 – -0.22) |
| High Probability condition : Trial | 0.27  (-0.80 – 1.33) | 0.02  (-0.06 – 0.09) |
| High Probability condition : Trial : Aware group | -2.47  (-4.03 – -0.91) | -0.17  (-0.28 – -0.06) |
| High Probability condition | -0.34  (-1.00 – 0.33) | -0.05  (-0.14 – 0.04) |
| Zero Probability condition | 0.14  (-0.52 – 0.81) | 0.08  (-0.02 – 0.18) |
| Zero Probability condition: Aware group | 0.41  (-0.57 – 1.39) | 0.06  (-0.09 – 0.20) |
| Zero Probability condition: Trial | 0.40  (-0.65 – 1.45) | 0.03  (-0.04 – 0.10) |
| Zero Probability condition: Trial : Aware group | -0.32  (-1.86 – 1.22) | -0.02  (-0.13 – 0.08) |
| Unaware group | *Reference* |  |
| Aware group | -0.61  (-1.49 – 0.28) | -0.20  (-0.37 – -0.04) |
| Trial | -0.50  (-1.25 – 0.24) | -0.03  (-0.09 – 0.02) |
| Trial : Aware group | -0.46  (-1.56 – 0.63) | -0.03  -0.11 – 0.04 |
| ICC | 0.08 |  |
| Marginal R^2^ / Conditional R^2^ | 0.06 / 0.13 |  |

### Table S9. CNV amplitude slope over trials – contrast analysis

| Group | Slope | | | Contrast | df | *t*-score | *p-*value |
| --- | --- | --- | --- | --- | --- | --- | --- |
|  | **High Probability** | **Low Probability** | **Zero Probability** |  |  |  |  |
| Aware | -3.171 | -0.966 | -0.887 | **Low-High** | 6786 | 3.770 | <0.001 |
|  |  |  |  | **Low-Zero** | 6787 | - 0.138 | 0.990 |
|  |  |  |  | **High-Zero** | 6787 | - 3.917 | <0.001 |
| Unaware | -0.236 | -0.502 | -0.100 | **Low-High** | 6810 | -0.489 | 0.877 |
|  |  |  |  | **Low-Zero** | 6792 | -0.751 | 0.733 |
|  |  |  |  | **High-Zero** | 6806 | -0.252 | 0.966 |
| Degrees-of-freedom method: Satterthwaite, *p*-value adjustment method: Tukey | | | | | | | |

Target-evoked P300 (P3b)

An LMM was fitted to single-trial ERP amplitudes of standard and target trials measured within the P3b region of interest. Due to low number of targets occurring in Low Probability condition, only trials from High Probability condition were used. Mild a priori data trimming (<1%) yielded a dataset consisting of 8833 observations. The initial model formulated in R syntax was: P300 amplitude ~ stimulus*trial*group + (stimulus | subject). Step-wise model reduction procedure revealed that (a) the optimal model includes random intercept and slopes for stimulus over subjects, and (b) the optimal model does not include the following terms: stimulus:trial:group, trial:group, stimulus:group, and group (see Table S10). Thus, the final model was more parsimonious than the initial model and did not indicate group differences in the P3b component. Its formula according to R syntax was: P300 amplitude ~ stimulus + trial + stimulus:trial + (stimulus | subject). A satisfactory distribution of model residuals was achieved by further removing data points associated with extreme residuals. The final model was thus fitted with 8784 observations (98.5% of the original dataset).

### Table S10. Target-P300 amplitude model selection

| Model | Eliminated term | AIC | BIC | LL | Dev. | χ^2^ | df | *p* |  |
| --- | --- | --- | --- | --- | --- | --- | --- | --- | --- |
| Initial | none | 45238.8 | 45323.8 | -22607.4 | 45214.8 |  |  |  |  |
| Step 1 | Random slope by stimulus | 45661.7 | 45732.5 | -22820.8 | 45641.7 | 426.91 | 2 | <0.001 |  |
| Step 2 | Group : stimulus : trial | 45240.2 | 45318.1 | -22609.1 | 45218.2 | 3.41 | 1 | 0.065 |  |
| Step 3 | Group : trial | 45238.3 | 45309.2 | -22609.2 | 45218.3 | 0.14 | 1 | 0.708 |  |
| Step 4 | Stimulus : group | 45236.5 | 45300.3 | -22609.3 | 45218.5 | 0.22 | 1 | 0.639 |  |
| Step 5 | Group | 45235.6 | 45292.3 | -22609.8 | 45219.6 | 1.04 | 1 | 0.308 |  |
| Step 6 | Stimulus : trial | 45241.5 | 45291.1 | -22613.8 | 45227.5 | 7.97 | 1 | 0.005 |  |
| Underscored terms were retained | | | | | | | | | |

*
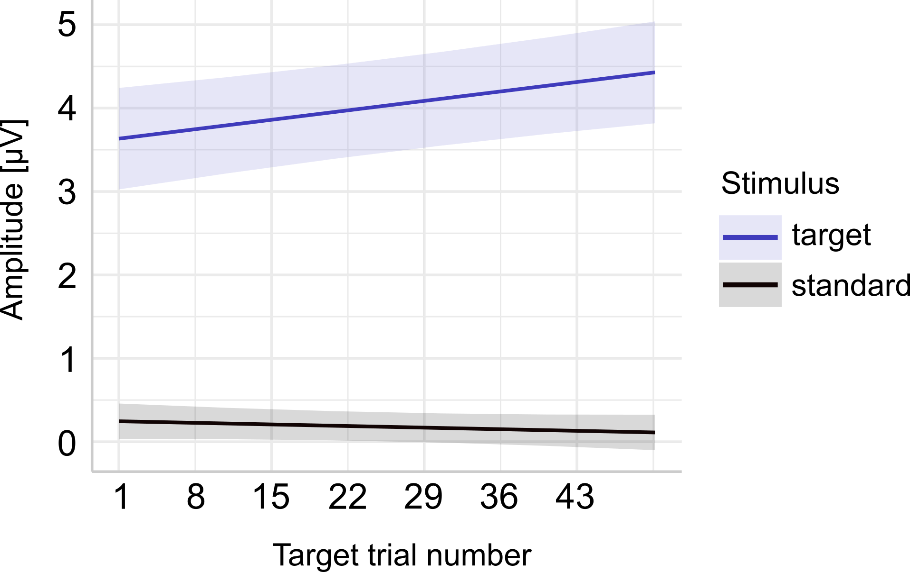
*

### Figure S3. Target-P300 amplitude slopes over trials for standards and targets

Fig. S3 shows LMM predictions of the P300 amplitudes. Shaded areas indicate 95% confidence intervals.

### Table S11. Target-P300 amplitude estimates for standards and targets

| **Predictors** | **Estimates**  **(CI)** | **std. Beta**  **(standardized CI)** |
| --- | --- | --- |
| (Intercept) | 0.25  (0.03 – 0.46) | -0.22  (-0.27 – -0.18) |
| Standard stimulus | *Reference* |  |
| Target stimulus | 3.39  (2.76 – 4.02) | 1.09  (0.93 – 1.25) |
| Target stimulus : Trial | 0.93  (0.39 – 1.47) | 0.08  (0.03 – 0.12) |
| Trial | -0.13  (-0.38 – 0.11) | -0.01  (-0.03 – 0.01) |
| ICC | 0.10 |  |
| Marginal R^2^ / Conditional R^2^ | 0.196 / 0.277 |  |

## Estimation of neural sources

Two source models were constructed: first (MODEL 1), to investigate the brain sources of cue-evoked ERPs related to contingency learning (therefore based on aware learners’ data from the oddball-SL task); second (MODEL 2), to investigate the influence of contingency learning and contingency awareness on brain sources of the P3b (therefore based on datasets from all subjects and both tasks).

After constructing the models, the contribution of each source (i.e. IC cluster) to the relevant ERP component was assessed by calculating the ‘percentage of variance accounted for’ (PVAF;^4^, which informs us how much EEG signal variance across channels can be explained by a given source relative to the remaining sources.

Finally, the activation of each IC within a given source is back-projected from its source to the scalp sensors. Summed IC projections originating from a given cluster are root-mean-squared across all channels yielding source-resolved ERP traces. A non-parametric permutation test (N = 10000) was performed on mean source-resolved ERP amplitudes between High and Low Probability cues (source model 1) or targets and standards (source model 2). In the case of model 1, these tests were performed in each time window (450-600 ms and 650-900 ms), whereas in model 2 – separately in each group, both tasks, and within the oddball-SL task – two phases. Significance levels were adjusted for multiple comparisons using the Bonferroni-Holm method.

MODEL 1 (CUE-RELATED)

The effective sources consisted of 11 to 31 independent components. The number of unique subjects who contributed independent components to a given source ranged from 11 to 21 (out of 22 aware learners). MNI coordinates of the clusters’ centroids were converted into the Talairach coordinate system^5^, and the Talairach Client^6^ was used to determine brain region labels.

### Table S12. Independent component clusters – model 1

| **Source** | **N_IC_** | **N_S_** | **MNI**  **coordinates** | **d  [mm]** | **Lobe**  **(hemisphere)** | **Source label** |
| --- | --- | --- | --- | --- | --- | --- |
| S1 | 23 | 16 | 40 -68 10 | 3 | Occipital (R) | Middle Occipital Gyrus  (BA 19) |
| S2 | 18 | 13 | -59 -2 7 | 1 | Frontal (L) | Precentral Gyrus  (BA 6) |
| S3 | 25 | 16 | -26 -16 52 | 2 | Frontal (L) | Precentral Gyrus  (BA 6) |
| S4 | 31 | 19 | -4 -6 31 | 0 | Limbic (L) | Cingulate Gyrus  (BA 24) |
| S5 | 24 | 17 | 41 -19 58 | 1 | Frontal (R) | Precentral Gyrus  (BA 4) |
| S6 | 21 | 15 | -18 -98 -12 | 2 | Occipital (L) | Lingual Gyrus  (BA 17) |
| S7 | 16 | 12 | 44 21 7 | 1 | Frontal (R) | Inferior Frontal Gyrus  (BA 13) |
| S8 | 22 | 15 | -3 -64 52 | 0 | Parietal (L) | Precuneus  (BA 7) |
| S9 | 15 | 12 | -29 -77 19 | 4 | Occipital (L) | Middle Occipital Gyrus  (BA 19) |
| S10 | 21 | 16 | -55 -54 3 | 5 | Temporal (L) | Superior Temporal Gyrus  (BA 22) |
| S11 | 20 | 14 | -38 -41 53 | 0 | Parietal (L) | Inferior Parietal Lobule  (BA 40) |
| S12 | 31 | 21 | 7 -74 19 | 0 | Occipital (R) | Cuneus  (BA 18) |
| S13 | 11 | 11 | 25 -91 -12 | 1 | Occipital (R) | Inferior Occipital Gyrus  (BA 18) |
| S14 | 23 | 14 | -33 -2 53 | 0 | Frontal (L) | Middle Frontal Gyrus  (BA 6) |
| S15 | 26 | 15 | 4 -33 67 | 0 | Frontal (R) | Paracentral Lobule  (BA 6) |
| S16 | 17 | 12 | 64 -26 20 | 1 | Parietal (R) | Postcentral Gyrus  (BA 40) |
| Notes: N_IC_ – number of independent components (ICs) within a given source; N_S_ – number of unique subjects contributing ICs to this source; MNI – x, y, z brain coordinates of the Montreal Neurological Institute; d – distance from the source centroid to the closest gray matter region; L – left hemisphere, R – right hemisphere; BA – Brodmann area | | | | | | |

### Table S13. Sources’ contributions to signal variance – model 1

| **Source** | **Source label** | **PVAF [%]**  **(400-650 ms)** | **PVAF [%]**  **(650-900 ms)** |
| --- | --- | --- | --- |
| S1 | Middle Occipital Gyrus (BA 19) | 15.3 | 17.5 |
| S2 | Precentral Gyrus (BA 6) | 1.9 | -4.2 |
| **S3** | **Precentral Gyrus (BA 6)** | 7.8 | **23.8** |
| **S4** | **Anterior Cingulate Cortex (BA 24)** | -5.1 | **39.6** |
| S5 | Precentral Gyrus (BA 4) | -11.0 | -2.9 |
| S6 | Lingual Gyrus (BA 17) | 13.0 | 9.8 |
| S7 | Inferior Frontal Gyrus (BA 13) | 13.9 | 12.6 |
| **S8** | **Precuneus (BA 7)** | **32.0** | 3.0 |
| **S9** | **Middle Occipital Gyrus (BA 19)** | **34.1** | 16.8 |
| S10 | Superior Temporal Gyrus (BA 22) | -0.1 | 16.4 |
| S11 | Inferior Parietal Lobule (BA 40) | 5.1 | -5.6 |
| S12 | Cuneus (BA 18) | -11.6 | -20.2 |
| **S13** | **Inferior Occipital Gyrus (BA 18)** | **29.2** | **29.0** |
| S14 | Middle Frontal Gyrus (BA 6) | 10.9 | 13.2 |
| S15 | Paracentral Lobule (BA 6) | 9.8 | -12.4 |
| S16 | Postcentral Gyrus (BA 40) | -7.3 | -1.3 |
| Notes: PVAF – ‘percentage of variance accounted for’ – a measure of signal variance within a given time window that can be attributed to a given source activity. Primary sources in bold | | | |

### Table S14. Statistical tests for ERPs elicited by model 1 primary sources

|  | **Source** | **Source label** |  | **t** | **df** | ***p*-value** |
| --- | --- | --- | --- | --- | --- | --- |
| 400-650  ms | S3 | Precentral Gyrus (BA-6) |  | 0.37 | 24 | 0.722 |
|  | S4 | Anterior Cingulate Cortex (BA-24) |  | -1.54 | 30 | 0.136 |
|  | S 8 | Precuneus (BA-7) |  | 3.17 | 21 | 0.002 * |
|  | S 9 | Middle Occipital Gyrus (BA-19) |  | 1.45 | 14 | 0.170 |
|  | S 13 | Inferior Occipital Gyrus (BA-18) |  | -0.60 | 10 | 0.576 |
| 650-900  ms | S 3 | Precentral Gyrus (BA-6) |  | -0.77 | 24 | 0.455 |
|  | S 4 | Anterior Cingulate Cortex (BA-24) |  | -3.03 | 30 | 0.003 * |
|  | S 8 | Precuneus (BA-7) |  | 1.63 | 21 | 0.115 |
|  | S 9 | Middle Occipital Gyrus (BA-19) |  | 1.46 | 14 | 0.161 |
|  | S 13 | Inferior Occipital Gyrus (BA-18) |  | 0.26 | 10 | 0.802 |
| Notes: results of a permutation test; asterisk indicate significance after the Bonferroni-Holm correction (*, **, and *** correspond to alpha level <0.05, <0.01, <0.001, respectively) | | | | | | |

MODEL 2 (TAREGT-RELATED)

The effective sources consisted of 36 to 133 independent components. The number of unique subjects who contributed independent components to a given source ranged from 27 to 47 (out of 48). Both groups contributed to every source proportionally to the ratio of aware to unaware subjects, i.e. on average 45% (SD = 3.3%) of the independent components originated from aware subjects. In case of the learning task, primary sources were determined separately for each phase of the SL-task (initial, late). MNI coordinates of the clusters’ centroids were converted into Talairach coordinate system^5^ and the Talairach Client^6^) was used to determine brain region labels.

### Table S15. Independent component clusters – model 2

| **Source** | **N_IC_** | **N_S_** | **MNI coordinates** | **d**  **[mm]** | **Lobe**  **(hemisphere)** | **Source label** |
| --- | --- | --- | --- | --- | --- | --- |
| S1 | 117 (54) | 44 (20) | -1 -49 58 | 1 | Parietal (L) | Precuneus  (BA 7) |
| S2 | 108 (46) | 43 (19) | 34 -76 15 | 5 | Occipital (R) | Middle Occipital Gyrus  (BA 19) |
| S3 | 133 (65) | 44 (20) | -1 -7 39 | 1 | Limbic (L) | Anterior Cingulate Cortex  (BA 24) |
| S4 | 106 (47) | 40 (18) | 7 -45 21 | 3 | Limbic (R) | Posterior Cingulate Cortex  (BA 23) |
| S5 | 79 (34) | 38 (17) | 61 -45 5 | 0 | Temporal (R) | Superior Temporal Gyrus  (BA 22) |
| S6 | 36 (17) | 27 (13) | -55 16 10 | 0 | Frontal (L) | Precentral Gyrus  (BA 44) |
| S7 | 105 (43) | 43 (19) | -40 -62 7 | 4 | Temporal (L) | Middle Temporal Gyrus  (BA37) |
| S8 | 131 (62) | 47 (22) | -37 -23 53 | 0 | Frontal (L) | Precentral Gyrus  (BA 4) |
| S9 | 116 (61) | 46 (21) | -3 23 48 | 0 | Frontal (L) | Medial Frontal Gyrus  (BA 8) |
| S10 | 69 (32) | 37 (16) | 23 -91 -15 | 1 | Occipital (R) | Inferior Occipital Gyrus  (BA 18) |
| S11 | 82 (39) | 40 (18) | -21 -97 -11 | 2 | Occipital (L) | Lingual Gyrus  (BA 18) |
| S12 | 50 (24) | 31 (16) | -62 -30 9 | 1 | Temporal (L) | Superior Temporal Gyrus  (BA 42) |
| S13 | 40 (22) | 28 (14) | 19 41 15 | 0 | Frontal (R) | Medial Frontal Gyrus  (BA 10) |
| S14 | 63 (29) | 35 (14) | 53 8 18 | 1 | Frontal (R) | Inferior Frontal Gyrus  (BA 44) |
| S15 | 106 (47) | 44 (19) | 39 -20 56 | 1 | Frontal (R) | Precentral Gyrus  (BA 4) |
| S16 | 77 (42) | 38 (18) | -8 -81 32 | 0 | Occipital (L) | Cuneus (BA 19) |
| Notes: N_IC_ – number of independent components (ICs) within a given source; N_S_ – number of unique subjects contributing ICs to this source; Numbers in brackets indicate the aware subjects’ contributions to NIC and NS; MNI – x, y, z brain coordinates of the Montreal Neurological Institute; d – distance from the source centroid to the closest gray matter region; L – left hemisphere, R – right hemisphere; BA – Brodmann area | | | | | | |

### Table S16. Sources’ contributions to signal variance – model 2

|  |  | | **PVAF [%]** | | | | | | |  |  |  |  |
| --- | --- | --- | --- | --- | --- | --- | --- | --- | --- | --- | --- | --- | --- |
| **Source** | | **Source label** | | Unaware  Control task | Unaware  SL_C_, initial phase | Unaware  SL_C_, final phase | Aware control task | Aware  SL_C_, initial phase | Aware   SL_C_, final phase | |  |  |  |
| **S1** | | **Precuneus**  **(BA 7)** | | -7.2 | **30.5** | **31.7** | **34.2** | **26.5** | **17.7** | |  |  |  |
| S2 | | Middle Occipital Gyrus  (BA 19) | | -12.3 | 4.9 | 8.6 | 2.1 | 2.6 | 7.0 | |  |  |  |
| **S3** | | **Anterior Cingulate Cortex**  **(BA 24)** | | **21.3** | 16.9 | 16.8 | **42.1** | **60.6** | **76.4** | |  |  |  |
| **S4** | | **Posterior Cingulate Cortex**  **(BA 23)** | | 5.5 | **22.3** | **30.1** | 13.5 | 2.6 | -15.1 | |  |  |  |
| S5 | | Superior Temporal Gyrus  (BA 22) | | 2.5 | -0.3 | -5.8 | -1.5 | 3.7 | -3.3 | |  |  |  |
| S6 | | Precentral Gyrus  (BA 44) | | 1.8 | 4.5 | 2.7 | 14.9 | -1.4 | -9.5 | |  |  |  |
| S7 | | Middle Temporal Gyrus  (BA37) | | -12.8 | 14.7 | 4.0 | -19.7 | 1.8 | -1.0 | |  |  |  |
| S8 | | Precentral Gyrus  (BA 4) | | 9.9 | 15.2 | 12.2 | 2.6 | 7.1 | 11.4 | |  |  |  |
| S9 | | Medial Frontal Gyrus  (BA 8) | | 9.1 | 20.3 | 17.4 | 18.3 | 3.4 | 7.1 | |  |  |  |
| S10 | | Inferior Occipital Gyrus  (BA 18) | | -5.4 | 0.5 | -0.5 | 13.7 | 15.7 | 9.4 | |  |  |  |
| S11 | | Lingual Gyrus  (BA 18) | | -6.9 | -5.6 | -6.4 | 7.3 | -2.3 | 0.8 | |  |  |  |
| **S12** | | **Superior Temporal Gyrus**  **(BA 42)** | | **18.0** | -0.6 | 0.8 | -3.4 | 2.0 | -2.2 | |  |  |  |
| S13 | | Medial Frontal Gyrus  (BA 10) | | 7.5 | 2.1 | 6.2 | -7.3 | -2.8 | 2.9 | |  |  |  |
| S14 | | Inferior Frontal Gyrus  (BA 44) | | -2.5 | 3.3 | 5.1 | -2.5 | -1.9 | 0.9 | |  |  |  |
| S15 | | Precentral Gyrus  (BA 4) | | -13.2 | 14.6 | 12.5 | 9.6 | 11.4 | 10.0 | |  |  |  |
| **S16** | | **Cuneus**  **(BA 19)** | | **29.5** | **35.8** | **39.9** | **18.4** | **16.1** | **13.3** | |  |  |  |
|  | | | | | | | | | | | |  |  |

### Table S17. Statistical tests for ERPs elicited by model 2 primary sources

|  |  | **Brain region (Brodmann area)** | **t** | **df** | ***p*-value** |  |
| --- | --- | --- | --- | --- | --- | --- |
| Unaware  control task | S 1 | Precuneus (BA 7) | 5.14 | 30 | <0.001 *** |  |
|  | S 3 | Anterior Cingulate Cortex (BA 24) | 1.29 | 42 | 0.204 |  |
|  | S 4 | Posterior Cingulate Cortex (BA 23) | 1.24 | 26 | 0.228 |  |
|  | S 12 | Superior Temporal Gyrus (BA 42) | -1.40 | 8 | 0.196 |  |
|  | S 16 | Cuneus (BA 19) | 2.84 | 17 | 0.011 |  |
| Unaware  SL_C_, initial phase | S 1 | Precuneus (BA 7) | 4.73 | 31 | <0.001 *** |  |
|  | S 3 | Anterior Cingulate Cortex (BA 24) | 1.25 | 24 | 0.231 |  |
|  | S 4 | Posterior Cingulate Cortex (BA 23) | 2.57 | 31 | 0.016 |  |
|  | S 12 | Superior Temporal Gyrus (BA 42) | -0.98 | 16 | 0.352 |  |
|  | S 16 | Cuneus (BA 19) | 2.48 | 16 | 0.001 * |  |
| Unaware  SL_C_, final phase | S 1 | Precuneus (BA 7) | 6.02 | 31 | <0.001 *** |  |
|  | S 3 | Anterior Cingulate Cortex (BA 24) | 1.74 | 24 | 0.096 |  |
|  | S 4 | Posterior Cingulate Cortex (BA 23) | 1.93 | 31 | 0.064 |  |
|  | S 12 | Superior Temporal Gyrus (BA 42) | -3.34 | 16 | 0.005 |  |
|  | S 16 | Cuneus (BA 19) | 3.07 | 16 | <0.001 *** |  |
| Aware control task | S 1 | Precuneus (BA 7) | 3.80 | 24 | 0.001 * |  |
|  | S 2 | Anterior Cingulate Cortex (BA 24) | 0.40 | 30 | 0.708 |  |
|  | S 4 | Posterior Cingulate Cortex (BA 23) | 0.81 | 26 | 0.462 |  |
|  | S 12 | Superior Temporal Gyrus (BA 42) | -0.82 | 9 | 0.459 |  |
|  | S 16 | Cuneus (BA 19) | 2.25 | 19 | 0.027 |  |
| Aware SL_C_, initial phase | S 1 | Precuneus (BA 7) | 4.20 | 32 | <0.001** |  |
|  | S 3 | Anterior Cingulate Cortex (BA 24) | 3.74 | 33 | <0.001* |  |
|  | S 4 | Posterior Cingulate Cortex (BA 23) | 1.02 | 18 | 0.341 |  |
|  | S 12 | Superior Temporal Gyrus (BA 42) | -1.96 | 13 | 0.054 |  |
|  | S 16 | Cuneus (BA 19) | 1.54 | 21 | 0.138 |  |
| Aware SL_C_, final phase | S 1 | Precuneus (BA 7) | 3.24 | 28 | 0.002* |  |
|  | S 3 | Anterior Cingulate Cortex (BA 24) | 4.94 | 33 | <0.001*** |  |
|  | S 4 | Posterior Cingulate Cortex (BA 23) | -0.11 | 19 | 0.921 |  |
|  | S 12 | Superior Temporal Gyrus (BA 42) | -1.26 | 13 | 0.262 |  |
|  | S 16 | Cuneus (BA 19) | -0.40 | 21 | 0.696 |  |
| Notes: results of a permutation test; asterisk indicate significance after the Bonferroni-Holm correction (*, **, and *** correspond to alpha level <0.05, <0.01, <0.001, respectively) | | | | | | |
|  | | | | | |  |

## Additional results

The effects of task version and task order


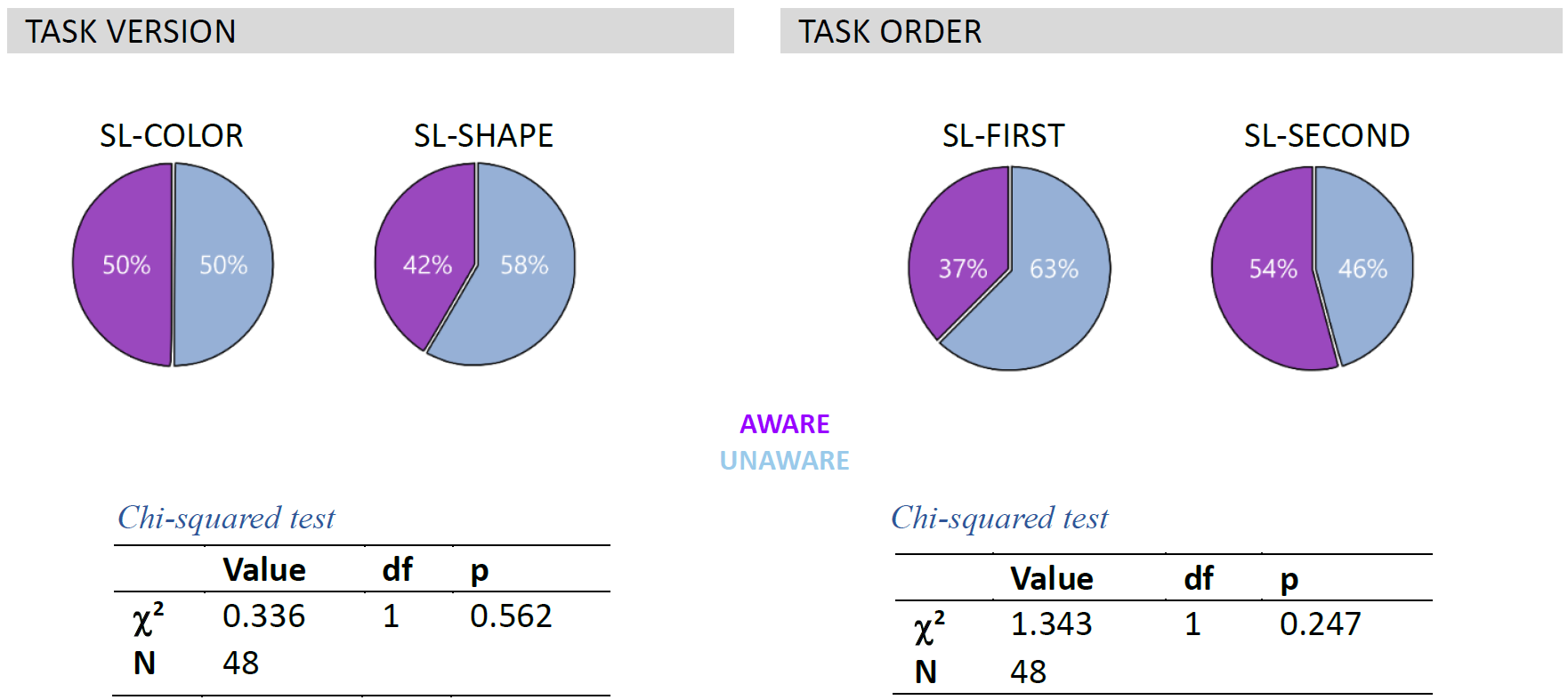


### Figure S4. Contingency awareness results – split by task version and task order


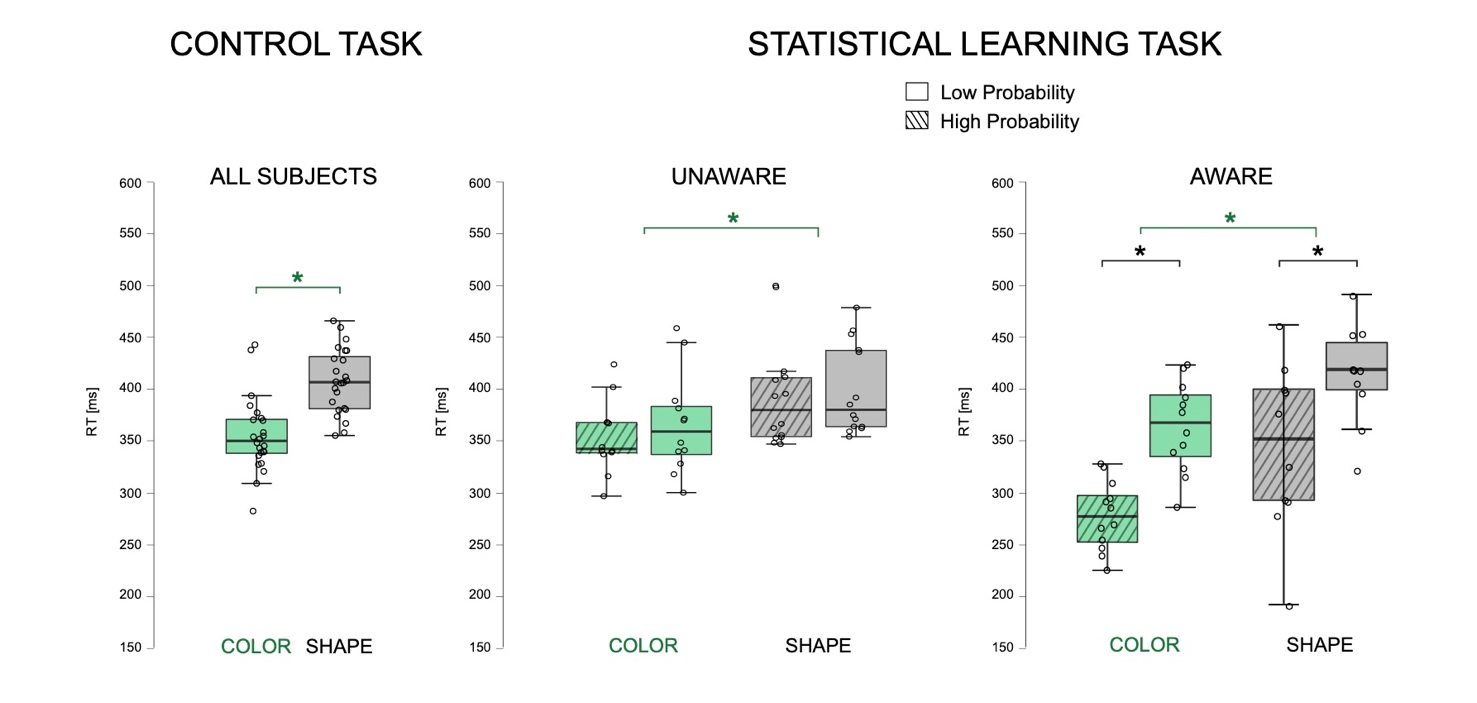


### Figure S5. Response times – split by task version


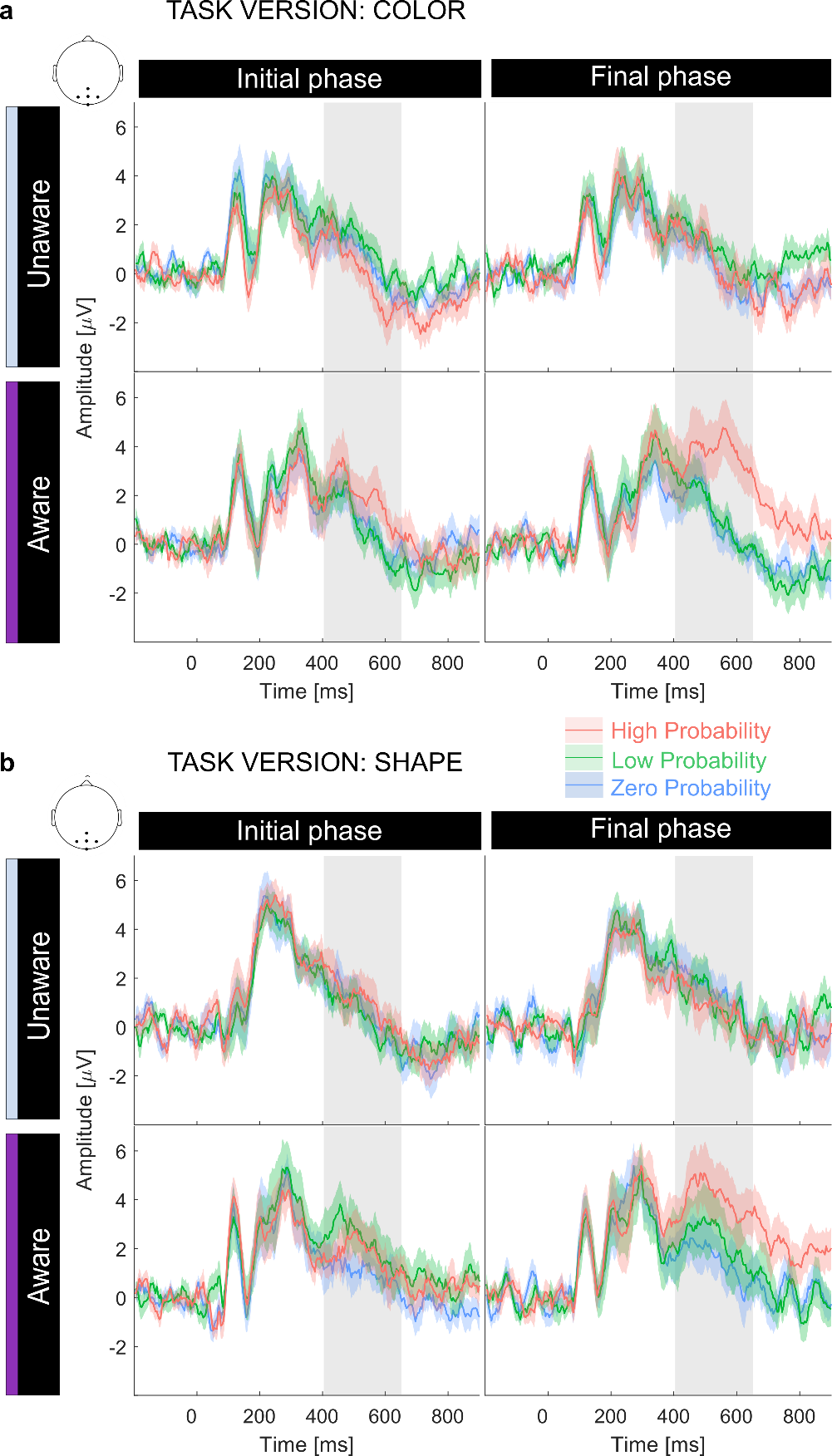


### Figure S6. Cue-P300 – split by task version


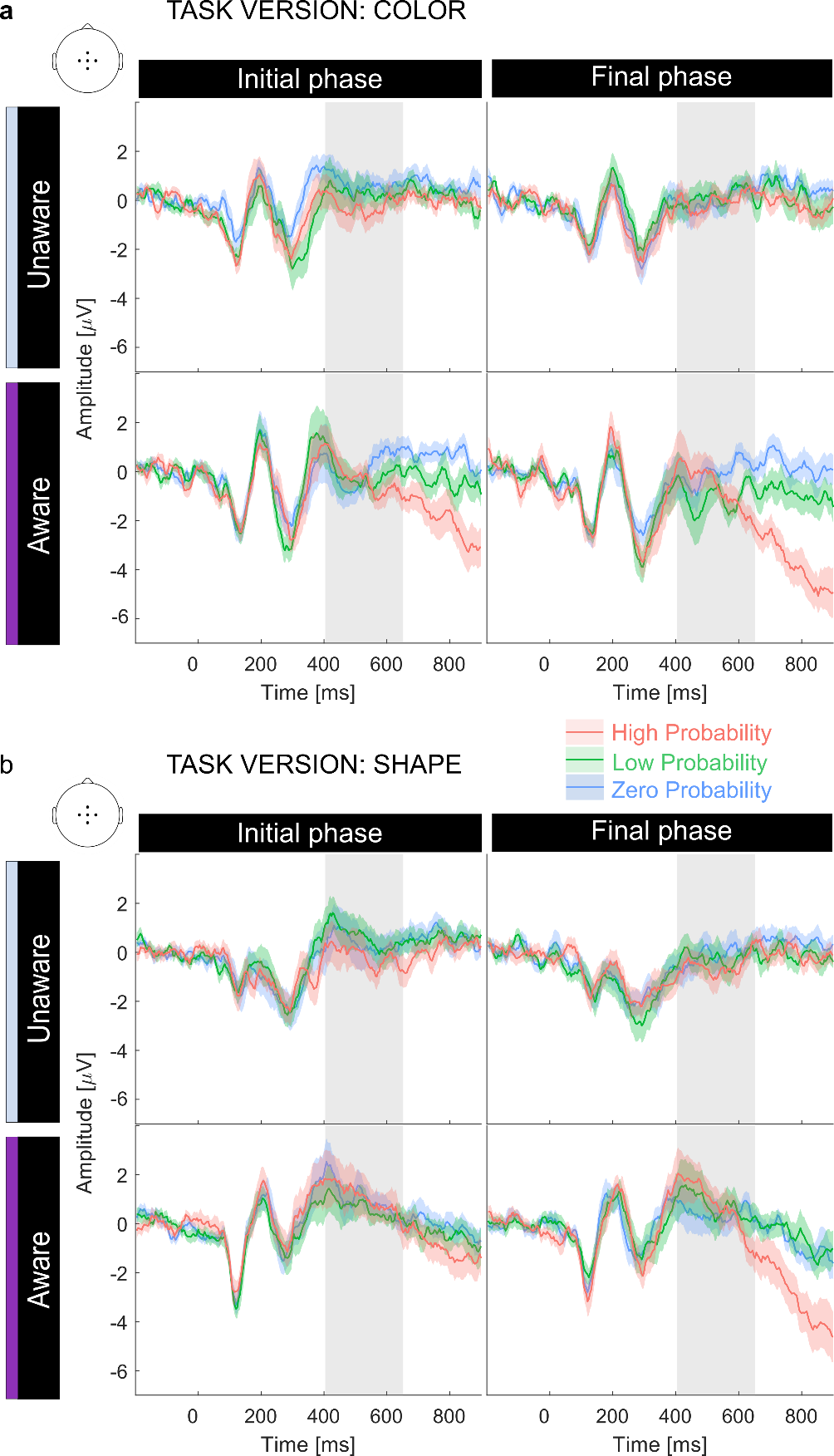


### Figure S7. CNV – split by task version

Classification of individual subject response times from the oddball-SL task

A linear regression model was fit to the RTs obtained in the oddball-SL task (lm() function from an R package stat). Independent variables were condition, trial, and subject. RT slopes over trials were estimated for each subject and condition (High, Low Probability) using the emtrends() function. Next, contrast between RT slopes in the two conditions were calculated for each subject, and the results were adjusted for multiple using the Satterthwaite approximation (Table S18).

### Table S18. Individual subject RT slopes – contrast analysis

| **Group** | **Subject** | **Slope (High P.)** | **Slope  (Low P.)** | **Contrast** | **SE** | **df** | **t-ratio** | **p-value** |
| --- | --- | --- | --- | --- | --- | --- | --- | --- |
| Aware | S01 | 0.02 | -0.06 | Low-High | 0.172 | 2633 | -0.46 | 0.643 |
| Aware | S02 | **-0.29** | 0.15 | Low-High | 0.188 | 2633 | 2.35 | **0.019** |
| Aware | S03 | **-0.29** | 0.08 | Low-High | 0.164 | 2633 | 2.25 | **0.025** |
| Aware | S04 | **-0.48** | 0.36 | Low-High | 0.161 | 2633 | 5.24 | **<0.001** |
| Aware | S05 | **-0.07** | 0.30 | Low-High | 0.18 | 2633 | 2.02 | **0.043** |
| Aware | S06 | **-0.55** | -0.01 | Low-High | 0.155 | 2633 | 3.46 | **0.001** |
| Aware | S07 | **-0.20** | 0.00 | Low-High | 0.153 | 2633 | 1.33 | 0.182 |
| Aware | S08 | **-0.17** | 0.22 | Low-High | 0.159 | 2633 | 2.47 | **0.014** |
| Aware | S09 | **-0.10** | -0.39 | Low-High | 0.155 | 2633 | -1.90 | 0.058 |
| Aware | S10 | **-0.13** | 0.14 | Low-High | 0.156 | 2633 | 1.71 | 0.088 |
| Aware | S11 | 0.01 | -0.30 | Low-High | 0.174 | 2633 | -1.80 | 0.073 |
| Aware | S12 | **-0.24** | -0.02 | Low-High | 0.169 | 2633 | 1.34 | 0.181 |
| Aware | S13 | **-0.56** | 0.38 | Low-High | 0.204 | 2633 | 4.63 | **<0.001** |
| Aware | S14 | 0.05 | 0.10 | Low-High | 0.153 | 2633 | 0.33 | 0.742 |
| Aware | S15 | **-0.51** | 0.65 | Low-High | 0.152 | 2633 | 7.64 | **<0.001** |
| Aware | S16 | **-0.77** | 0.31 | Low-High | 0.163 | 2633 | 6.63 | **<0.001** |
| Aware | S17 | **-0.58** | 0.11 | Low-High | 0.151 | 2633 | 4.60 | **<0.001** |
| Aware | S18 | **-0.36** | 0.33 | Low-High | 0.176 | 2633 | 3.91 | **<0.001** |
| Aware | S19 | **-0.18** | -0.30 | Low-High | 0.156 | 2633 | -0.77 | 0.441 |
| Aware | S20 | **-0.35** | -0.07 | Low-High | 0.184 | 2633 | 1.51 | 0.132 |
| Aware | S21 | **-0.22** | -0.15 | Low-High | 0.154 | 2633 | 0.47 | 0.638 |
| Aware | S22 | **-0.71** | 0.26 | Low-High | 0.203 | 2633 | 4.79 | **<0.001** |
| Unaware | S23 | **-0.20** | -0.01 | Low-High | 0.17 | 2633 | 1.11 | 0.267 |
| Unaware | S24 | 0.01 | -0.06 | Low-High | 0.171 | 2633 | -0.41 | 0.681 |
| Unaware | S25 | 0.22 | -0.19 | Low-High | 0.23 | 2633 | -1.76 | 0.079 |
| Unaware | S26 | 0.02 | -0.11 | Low-High | 0.155 | 2633 | -0.80 | 0.422 |
| Unaware | S27 | **-0.09** | 0.10 | Low-High | 0.147 | 2633 | 1.25 | 0.211 |
| Unaware | S28 | 0.02 | -0.43 | Low-High | 0.16 | 2633 | -2.85 | **0.004** |
| Unaware | S29 | **-0.19** | -0.39 | Low-High | 0.158 | 2633 | -1.27 | 0.203 |
| Unaware | S30 | **-0.04** | 0.02 | Low-High | 0.163 | 2633 | 0.33 | 0.741 |
| Unaware | S31 | 0.09 | -0.16 | Low-High | 0.152 | 2633 | -1.67 | 0.095 |
| Unaware | S32 | **-0.05** | -0.12 | Low-High | 0.162 | 2633 | -0.42 | 0.672 |
| Unaware | S33 | **-0.05** | 0.12 | Low-High | 0.156 | 2633 | 1.07 | 0.285 |
| Unaware | S34 | **-0.04** | 0.11 | Low-High | 0.163 | 2633 | 0.93 | 0.351 |
| Unaware | S35 | **-0.08** | -0.04 | Low-High | 0.156 | 2633 | 0.27 | 0.786 |
| Unaware | S36 | 0.03 | 0.28 | Low-High | 0.154 | 2633 | 1.64 | 0.102 |
| Unaware | S37 | 0.26 | 0.05 | Low-High | 0.152 | 2633 | -1.37 | 0.172 |
| Unaware | S38 | 0.02 | 0.14 | Low-High | 0.189 | 2633 | 0.65 | 0.519 |
| Unaware | S39 | **-0.06** | 0.25 | Low-High | 0.157 | 2633 | 1.95 | 0.051 |
| Unaware | S40 | 0.03 | 0.12 | Low-High | 0.165 | 2633 | 0.56 | 0.578 |
| Unaware | S41 | **-0.01** | -0.01 | Low-High | 0.159 | 2633 | 0.00 | 0.999 |
| Unaware | S42 | 0.14 | -0.07 | Low-High | 0.152 | 2633 | -1.41 | 0.159 |
| Unaware | S43 | 0.15 | -0.07 | Low-High | 0.175 | 2633 | -1.24 | 0.215 |
| Unaware | S44 | 0.10 | 0.00 | Low-High | 0.165 | 2633 | -0.60 | 0.551 |
| Unaware | S45 | 0.04 | -0.08 | Low-High | 0.161 | 2633 | -0.77 | 0.444 |
| Unaware | S46 | **-0.04** | -0.07 | Low-High | 0.178 | 2633 | -0.18 | 0.861 |
| Unaware | S47 | **-0.03** | -0.09 | Low-High | 0.162 | 2633 | -0.33 | 0.742 |
| Unaware | S48 | **-0.02** | 0.01 | Low-High | 0.163 | 2633 | 0.21 | 0.838 |
| Negative RT slopes in the High Probability condition and significant differences in RT slopes between the High and Low Probability conditions (p <0.05) are marked in bold. | | | | | | | | |

Subjects were classified as showing online behavioral adaptation only if their individual RT data met the following two criteria: (1) the RT slope in High Probability condition had to be negative, showing that the subjects decreased their RTs to predictable targets over time, and (2) the RT slops in High and Low Probability conditions had to be significantly different. Based on these criteria, 12 subjects showed evidence for behavioral adaptation to contingencies (S2-S6, S8, S13, S15-S18, S22). All of them belonged to the contingency-aware group.

### Figure S8. Individual subject RT estimates and their classification

Fig. S8 (beginning on the previous page) shows the estimated RT slopes (log-transformed values) over subsequent blocks of trials and their corresponding 95% confidence intervals. Asterisks indicate subjects whose RT data show online behavioral adaptation (i.e. meet both previously defined criteria). Letters in the bottom left denote task version (S = shape; C = colour).

Response times in the oddball-control task

The response times obtained in the oddball-control task (see Table S18) did not differ significantly between the contingency-aware and unaware group (Wilcoxon rank-sum test: U(22,26) = 547, z = 0.155, *p =* 0.877).

Accuracy in the oddball tasks

Hit rate (based on target trials) and false alarm rate (based on non-target trials) were calculated to assess accuracy. *Hits* were defined as target trials with a response occurring within 1000 ms after target onset. The percentage of hits within all target trials constituted the *hit rate*. By analogy, *false alarms* were defined as trials with non-target stimuli followed by a response within 1000 ms from the stimulus onset. *False alarm rate* was defined as a percentage of false alarms within all non-target trials.

In both oddball tasks, the accuracy was at a ceiling level, i.e. hit scores were equal or close to 100%, and false alarms were below 1% (see Table S19). Groups did not differ significantly in **hit rate** (Wilcoxon rank-sum test; oddball-control task: U(22,26) = 561, z = 0.505, *p =* 0.614); oddball-SL task: U(22,26) = 553.5, z = 0.317, *p =* 0.751. Groups also did not differ **in false alarm** rate in the oddball-control task alarms (Wilcoxon rank-sum test: U(22,26) = 563, z = 0.494, *p =* 0.621). In the oddball-SL task, on the other hand, the aware group committed more false alarms than the unaware group (Wilcoxon rank-sum test: U(22,26) = 519, z = -2.4372, *p =* 0.015). This difference is, however, hard to interpret as an effect related to contingency learning because it could not be stated that the aware subjects committed significantly more false alarms in the oddball-SL than in the oddball-control task (Wilcoxon signed rank test: U(22) = 125, Z= -0.049, *p =* 0.961).

### Table S19. Accuracy and RTs in oddball tasks

| Task | Group | Hit score  (median and IQR) | False alarms  (median and IQR) | Response times (median and IQR) |
| --- | --- | --- | --- | --- |
| Oddball-SL | Aware | 100.0 (3.2) % | 0.79 (0.8) % | 311 (111) ms* |
|  | Unaware | 99.2 (3.2) % | 0.32 (0.5) % | 355 (74) ms* |
| Oddball-control | Aware | 100.0 (3.7) % | 0.50 (1.4) % | 355 (64) ms |
|  | Unaware | 100.0 (3.7) % | 0.36 (0.7) % | 353 (68) ms |
| Note: IQR – inter-quartile range;  *Primary analyses of response times from the oddball-SL task were performed using a linear mixed-effects model and are reported in the results section of the main manuscript and in Tables S1-S3 | | | |  |

**Mass-univariate tests for RT-matched ERPs**

After matching the trials by their associated RTs (see the main manuscript – methods), a cluster-mass permutation test yielded no significant differences in cue-evoked ERPs between the High- and Low Probability condition. The lowest obtained *p*-values in each data portion and for different values of the ΔRT parameter are reported in Table S20. Although in the final phase for aware subjects, the *p*-value for 10 ms criterion trended toward significance (*p* = 0.07), the corresponding cluster covered very narrow window of latencies shorter than the ERPs of interest (i.e. around 260 ms). Due to the prevalent lack of significant clusters, the raster plots are not shown.

### Table S20. Cluster *p*-values after RT-matching

| **Data portion** | **ΔRT = 5 ms** | **ΔRT = 10 ms** | **ΔRT = 15 ms** |
| --- | --- | --- | --- |
| Unaware group, initial phase | 0.551 | 0.419 | 1.000 † |
| Unaware group, final phase | 0.523 | 0.168 | 0.167 |
| Aware group, initial phase | 0.628 | 0.070 | 0.123 |
| Aware group, final phase | 0.105 | 0.422 | 0.475 |
| Note: reported are the lowest *p*-values among all obtained cluster *p*-values;  † due to the use of Bonferroni correction, clusters can take *p*-values greater than 1 | | | |

**Mass-univariate tests for RT-contrasted ERPs**

As reported in the main manuscript, a cluster-mass permutation test on the contingency-unaware group data did not yield significant differences in cue-evoked ERPs within the High Probability condition, i.e., between the cues associated with fast and slow responses: all cluster *p-*values were equal or larger than 0.030 (initial phase) and 0.029 (final phase). Although these values would have been considered significant by a less conservative criterion, the relevant clusters occurred at different scalp sites and latencies than the enhancement of the cue-P300 and CNV. The RT-contrasted ERPs within the discussed ROIs largely overlapped (see Fig. S9 a).

Instead, in the contingency-aware group, significant ERP differences between High Probability cues associated with slow and fast responses were observed in both phases; in the initial phase, cluster *p*-values ranged from <0.001 to 0.004, and in the initial phase were <0.001. The ERPs obtained from the discussed regions of interest are shown in Fig. S9 b, and the entire analyzed time-sensor space with the obtained clusters is shown in Fig. S10.


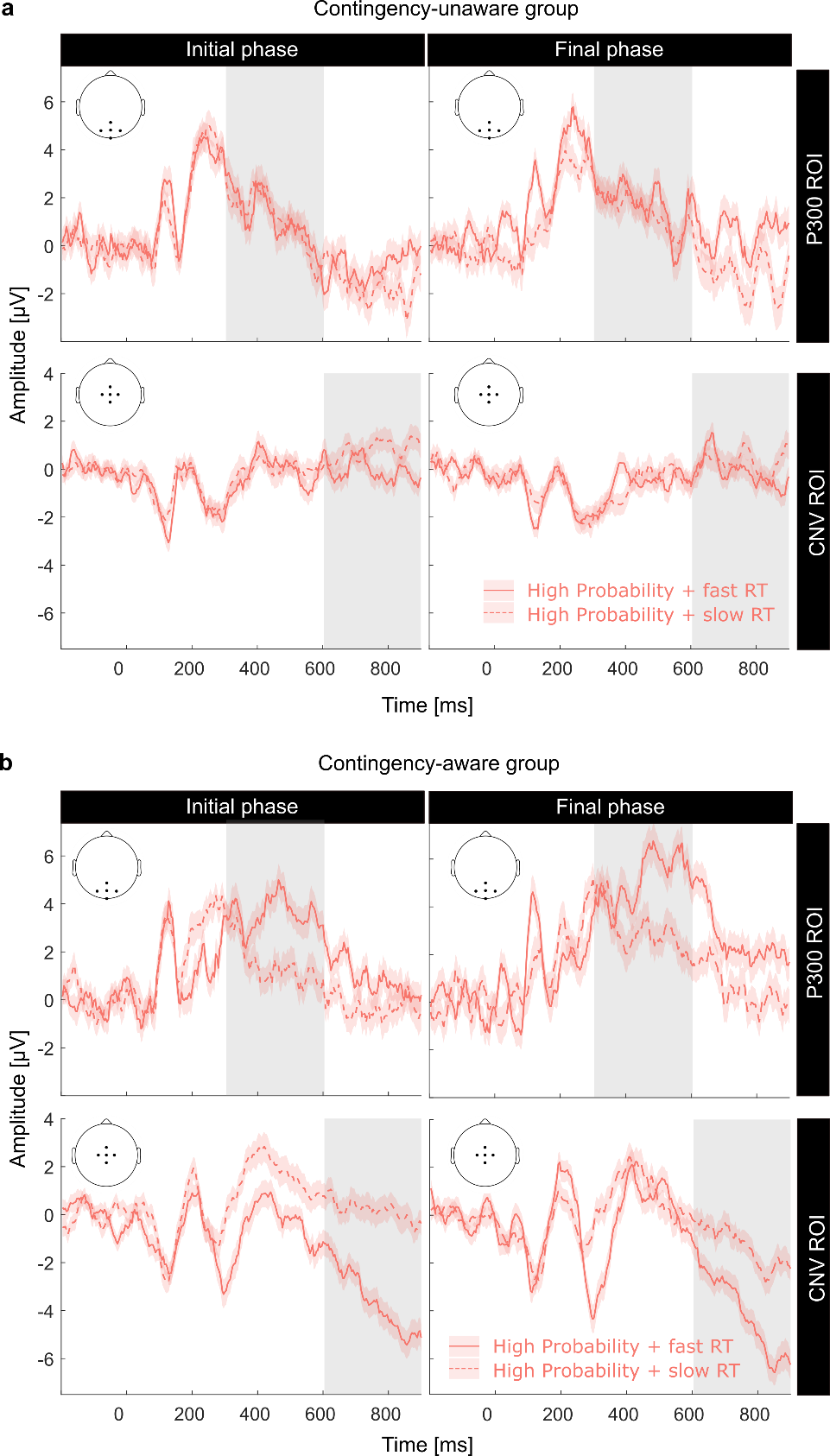


### Figure S9. Cue-ERPs after RT-contrasting


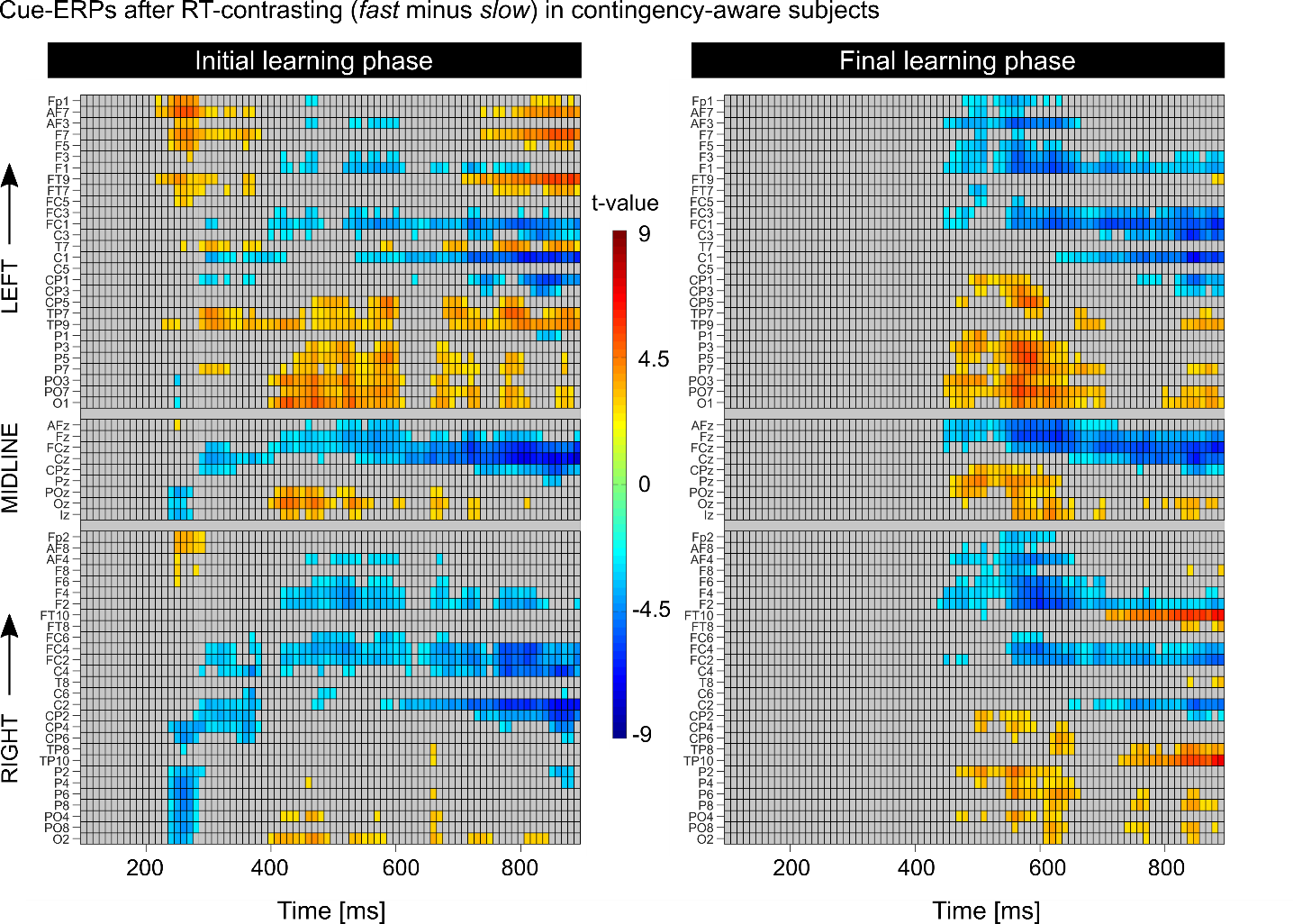


### Figure S10. Raster plots showing the result of RT-contrasting (aware group)

The raster plots in Fig. S10 present the results of cluster mass permutation tests. Only points within clusters associated with *p*-value < 0.01 are colored. Electrodes are represented as rows of these raster plots. The electrodes were organized topographically: left hemisphere electrodes on top, midline electrodes in the middle, and right hemisphere electrodes at the bottom (arrows on the left edge of the figure – indicate electrode organization from occipital to frontal sites within each electrode subgroup).

## Method details

The questionnaire probing explicit knowledge about contingencies

Immediately after completing the oddball-SL task, subjects responded to a questionnaire designed to probe their explicit knowledge about contingencies. The first three questions allowed the subject to spontaneously report their knowledge about the contingencies. If explicit knowledge was not revealed up to this point, the fourth question was critical for distinguishing between contingency-aware and contingency-unaware subjects. The remaining questions depended on the previous answer and aimed at probing the possible influence of implicit knowledge on post-exposure judgements (question 5) or probing the extent of the explicit knowledge (question 6). Subjects were classified as contingency-aware if they gained explicit knowledge about contingencies, i.e. if they reported, in their own words, the predictive relationship between the High Probability cue and the target.


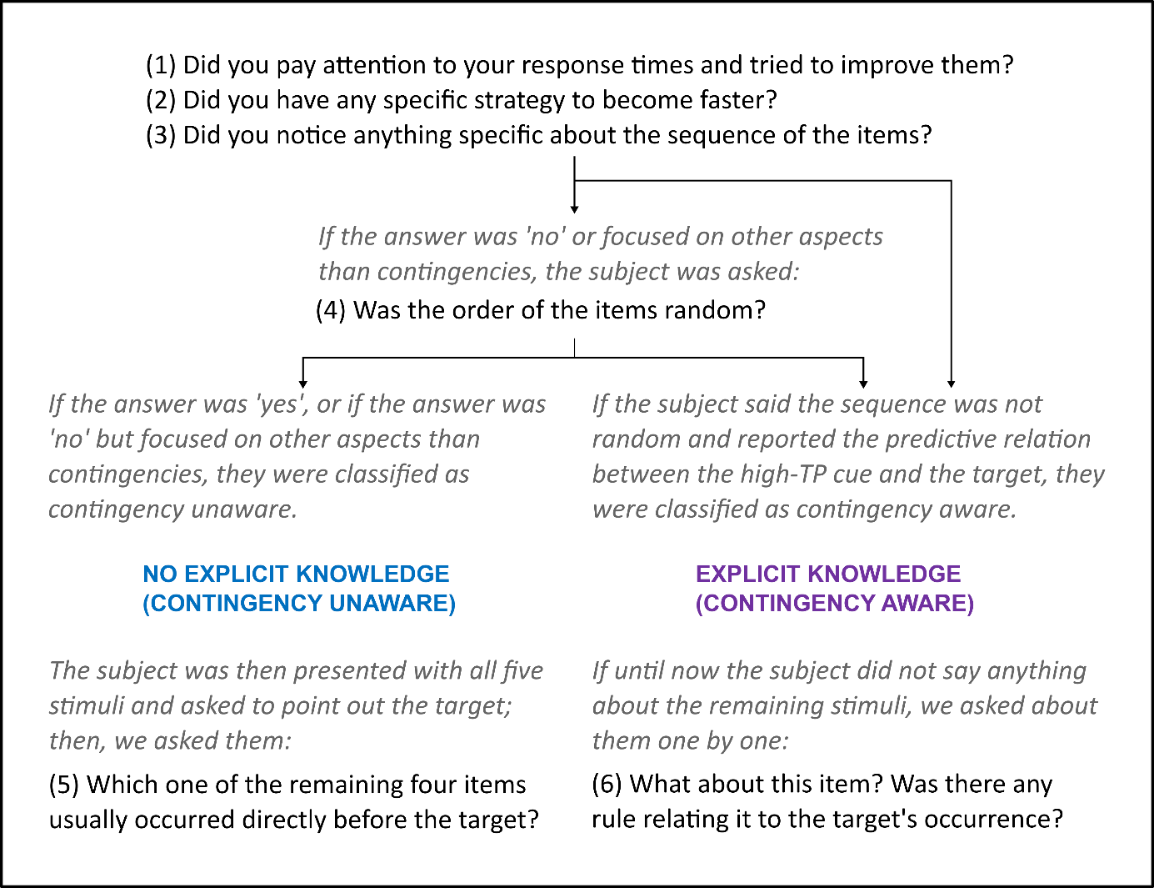


### Figure S11. Questionnaire

Determining ‘fast’ and ‘slow’ responses

To perform the RT-contrasting analysis of cue-evoked ERPs, the associated RTs had to be categorized into fast and slow. First, four data portions of the oddball-SL task were distinguished: two for the aware group (initial and late phase), and two for unaware (initial and late phase). Distributions of RTs within these data portions are presented in Fig. S12. 33% of the most extreme values on each side of the distribution constituted the fast (left-side), and slow (right-side) response category. The resultant category boundaries are provided in Table S21.

### Table S21. Boundaries of fast and slow RTs

|  | Fast | Slow |
| --- | --- | --- |
| Unaware, initial phase | 0 – 333 ms | 383 – 1000 ms |
| Unaware, final phase | 0 – 341 ms | 396 – 1000 ms |
| Aware, initial phase | 0 – 329 ms | 384 – 1000 ms |
| Aware, final phase | 0 – 225 ms | 262 – 1000 ms |


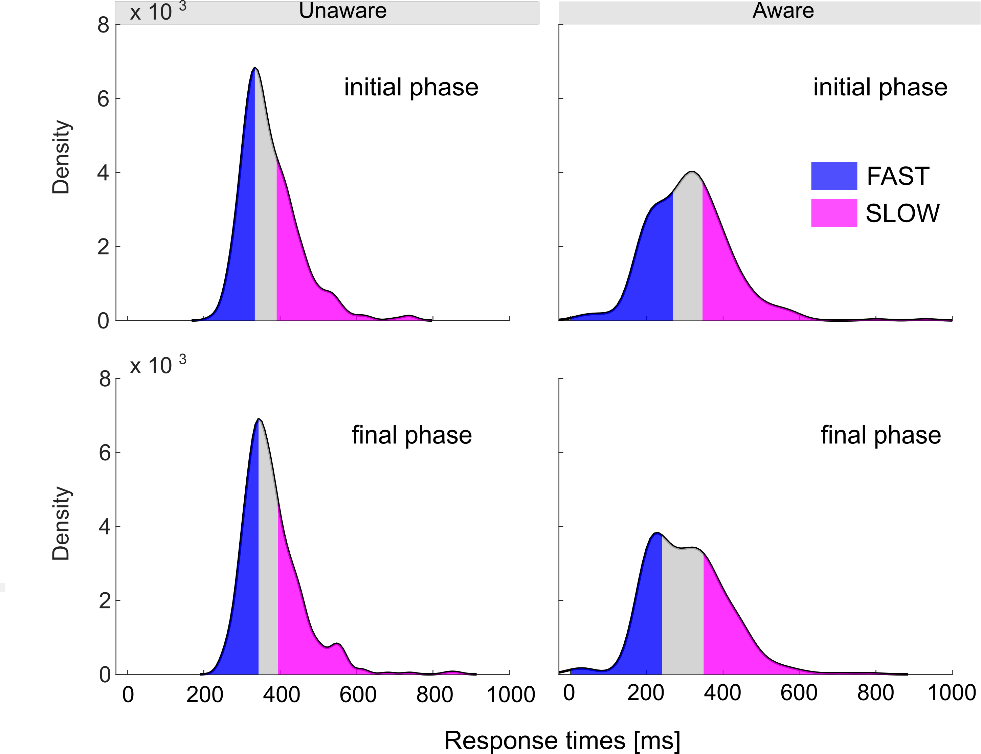


### Figure S12. RT distributions with fast and slow responses highlighted

### Table S22. Subjects’ demographics and task assignment

| **Subject** | **Age** | **Sex** | **Oddball-SL version** | **Tasks order** | **Answer to Question 5** | **Group** |
| --- | --- | --- | --- | --- | --- | --- |
| S02 | 31 | M | color | F-SL-OC | NA | AWARE |
| S05 | 26 | F | color | SL-OC-F | NA | AWARE |
| S06 | 23 | F | color | F-SL-OC | NA | AWARE |
| S07 | 18 | F | color | F-OC-SL | NA | AWARE |
| S08 | 30 | M | color | OC-SL-F | NA | AWARE |
| S09 | 27 | M | color | F-SL-OC | NA | AWARE |
| S11 | 21 | F | color | F-OC-SL | NA | AWARE |
| S14 | 20 | F | color | OC-SL-F | NA | AWARE |
| S18 | 32 | M | color | OC-SL-F | NA | AWARE |
| S20 | 22 | M | color | OC-SL-F | NA | AWARE |
| S21 | 20 | F | color | OC-SL-F | NA | AWARE |
| S22 | 20 | F | color | OC-SL-F | NA | AWARE |
| S10 | 25 | M | shape | F-SL-OC | NA | AWARE |
| S12 | 20 | F | shape | F-SL-OC | NA | AWARE |
| S13 | 23 | F | shape | F-OC-SL | NA | AWARE |
| S15 | 20 | M | shape | F-SL-OC | NA | AWARE |
| S16 | 29 | F | shape | F-OC-SL | NA | AWARE |
| S17 | 23 | F | shape | F-OC-SL | NA | AWARE |
| S19 | 27 | F | shape | OC-SL-F | NA | AWARE |
| S01 | 18 | M | shape | F-SL-OC | NA | AWARE |
| S03 | 18 | M | shape | F-OC-SL | NA | AWARE |
| S04 | 24 | M | shape | SL-OC-F | NA | AWARE |
| S24 | 22 | F | color | F-SL-OC | correct | UNAWARE |
| S25 | 36 | M | color | SL-OC-F | incorrect | UNAWARE |
| S29 | 28 | F | color | F-OC-SL | correct | UNAWARE |
| S31 | 20 | F | color | SL-OC-F | correct | UNAWARE |
| S32 | 23 | F | color | F-SL-OC | incorrect | UNAWARE |
| S36 | 27 | F | color | F-OC-SL | correct | UNAWARE |
| S40 | 27 | M | color | F-OC-SL | incorrect | UNAWARE |
| S41 | 30 | F | color | F-SL-OC | correct | UNAWARE |
| S43 | 20 | F | color | SL-OC-F | incorrect | UNAWARE |
| S44 | 29 | F | color | F-OC-SL | correct | UNAWARE |
| S45 | 25 | F | color | SL-OC-F | correct | UNAWARE |
| S47 | 37 | M | color | SL-OC-F | incorrect | UNAWARE |
| S23 | 31 | M | shape | SL-OC-F | incorrect | UNAWARE |
| S26 | 19 | M | shape | SL-OC-F | incorrect | UNAWARE |
| S27 | 21 | M | shape | SL-OC-F | incorrect | UNAWARE |
| S28 | 33 | M | shape | F-SL-OC | correct | UNAWARE |
| S30 | 23 | F | shape | OC-SL-F | correct | UNAWARE |
| S33 | 25 | F | shape | SL-OC-F | incorrect | UNAWARE |
| S34 | 22 | M | shape | F-SL-OC | correct | UNAWARE |
| S35 | 19 | F | shape | F-OC-SL | correct | UNAWARE |
| S37 | 23 | F | shape | OC-SL-F | incorrect | UNAWARE |
| S38 | 24 | M | shape | SL-OC-F | correct | UNAWARE |
| S39 | 26 | M | shape | OC-SL-F | incorrect | UNAWARE |
| S42 | 30 | M | shape | OC-SL-F | incorrect | UNAWARE |
| S46 | 20 | M | shape | OC-SL-F | incorrect | UNAWARE |
| S48 | 25 | F | shape | F-OC-SL | correct | UNAWARE |
| Legend for task order: F – flanker task, SL – oddball-SL, OC – oddball-control task | | | | | | |


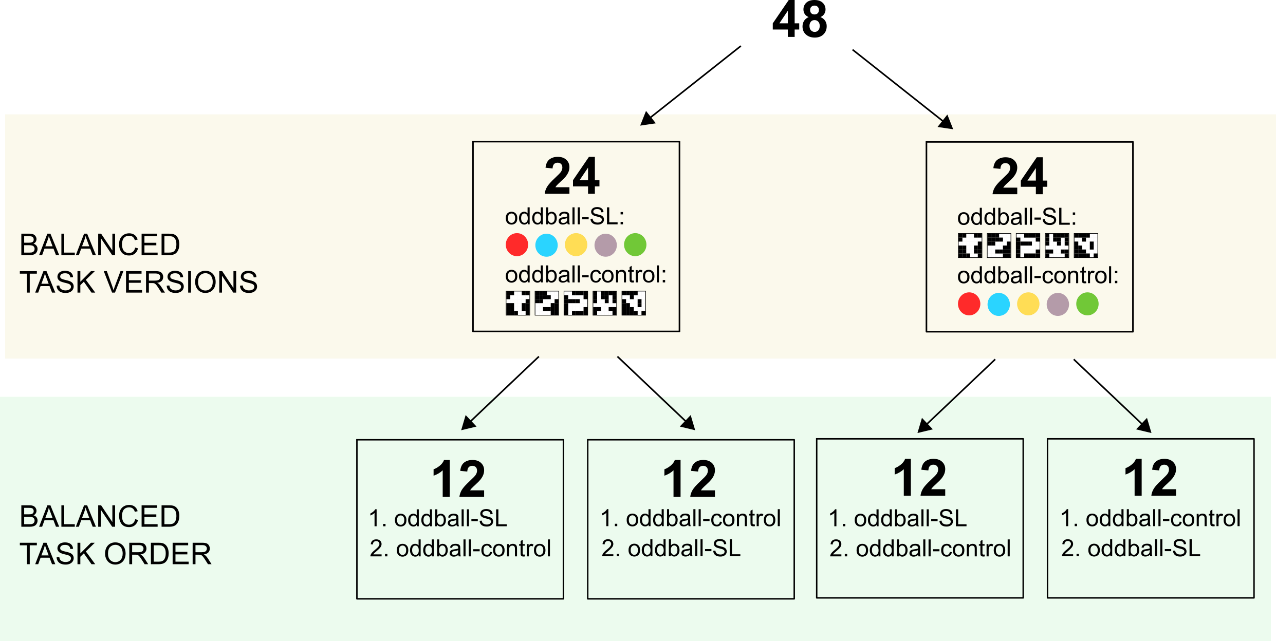


### Figure S13. Balanced assignment of task versions and order among participants

## References

1. Harald Baayen, R. & Milin, P. Analyzing reaction times. *Int. j. psychol. res.* **3**, 12–28 (2010).

2. Kuznetsova, A., Brockhoff, P. B. & Christensen, R. H. B. **lmerTest** Package: Tests in Linear Mixed Effects Models. *J. Stat. Soft.* **82**, (2017).

3. Lenth, R. V. Least-Squares Means: The *R* Package **lsmeans**. *J. Stat. Soft.* **69**, (2016).

4. Lee, C., Miyakoshi, M., Delorme, A., Cauwenberghs, G. & Makeig, S. Non-parametric group-level statistics for source-resolved ERP analysis. in *2015 37th Annual International Conference of the IEEE Engineering in Medicine and Biology Society (EMBC)* 7450–7453 (IEEE, 2015). doi:10.1109/EMBC.2015.7320114.

5. Brett, M., Christoff, K., Cusack, R. & Lancaster, J. Using the talairach atlas with the MNI template. *NeuroImage* **13**, 85 (2001).

6. Lancaster, J. L. *et al.* Automated Talairach Atlas labels for functional brain mapping. *Hum. Brain Mapp.* **10**, 120–131 (2000).
